# Dose-finding for dobutamine during transitional circulation in the very preterm infant: The study protocol

**DOI:** 10.1371/journal.pone.0338307

**Published:** 2025-12-19

**Authors:** Maria Sanchez-Holgado, Patricia Alvarez-Garcia, Maria Carmen Bravo, Fernando Cabañas, Manuela López Azorin, Izaskun Dorronsoro, Tania Carbayo Jimenez, Maria Maestro, Elena Diago-Sempere, Enrique Seco-Meseguer, María Jiménez-Gonzalez, Adelina Pellicer

**Affiliations:** 1 Department of Neonatology, La Paz University Hospital, Hospital La Paz Institute for Health Research-IdIPAZ, Madrid, Spain; 2 Department of Pediatrics, Quirónsalud Madrid University Hospital, Hospital La Paz Institute for Health Research-IdIPAZ, Madrid, Spain; 3 Neonatal Unit, Quirónsalud Madrid University Hospital, Madrid, Spain; 4 Department of Neonatology, 12 de Octubre Hospital, Madrid, Spain; 5 Clinical trials Unit, La Paz University Hospital, Madrid, Spain; PLOS: Public Library of Science, UNITED STATES OF AMERICA

## Abstract

**Background:**

Hemodynamic insufficiency occurs when tissue oxygen delivery fails to meet demand. Preterm infants are vulnerable to early circulatory failure after birth, impacting mortality and morbidity. Mean arterial pressure is not a reliable indicator of systemic blood flow. Low superior vena cava (SVC) flow, independent of fetal shunts, has been proposed as a robust biomarker of circulatory impairment in this population. Dobutamine, a selective β1 agonist with minimal effect on systemic vascular resistances, is considered the treatment of choice for this condition. However, information about its optimal dosing, efficacy, safety, and pharmacokinetic/pharmacodynamic (PK/PD) profile in preterm infants is limited.

**Methods:**

A multicenter, low-intervention, dose-finding trial will be conducted to establish the minimum effective dose of dobutamine to treat hemodynamic insufficiency, defined as SVC flow<51 mL/kg/min, in infants ≤32(+6) weeks of gestation during transitional circulation. Baseline echocardiography will be performed within 72 hours after birth. Single commercial dobutamine solution will be prepared and administered intravenously. Participants will be allocated to a dosage regimen by the study statistician, who will adjust probabilities of success based on therapeutic responses. Allocation will be guided by the dose with posterior probability closest to target (80%). PK/PD assessment will be done at 60 minutes after start of allocated dose and at 180 min of effective dobutamine dose infusion (that achieving stable SVC flow > 55 mL/kg/min). The proportion of patients achieving and maintaining adequate hemodynamic status with dobutamine alone during the first 72 hours (efficacy), side effects, and variables explaining interindividual PK/PD variability will be explored.

**Discussion:**

This study aims to define the minimum dose of dobutamine with a relevant PD effect on circulation in infants with hemodynamic insufficiency during transitional circulation. This is the first step towards future randomized clinical trials on efficacy and safety of dobutamine for the proposed indication.

**Trial registration:**

EU Clinical trials register, EU CT 2023-504915-34-00, registered 18 April 2023.

## Introduction

Hemodynamic insufficiency occurs when there is a state of poor organ blood flow resulting in cellular energy failure due to an inability of tissue oxygen delivery to satisfy tissue oxygen demand. Hemodynamic insufficiency after birth is commonly seen in babies born prematurely and this condition has a significant clinical impact. Epidemiologic studies indicate an association between hemodynamic abnormalities in the days after birth in the preterm infant and adverse outcomes [1–4].

The first hours after birth differ markedly from all other periods in human life and are characterized by a unique and dynamically changing anatomy. This is accompanied by striking changes in function as the circulatory system of the healthy newborn adapts rapidly to life outside the womb. However, a significant proportion of babies born at less than 32 weeks’ gestation require cardiovascular treatment in the hours after birth, even in the absence of identifiable pathologies [5,6]. This indicates that hemodynamic insufficiency in this age group can be related to an immature adaptation to birth, and the reason for that relies on [4,7–9]: 1) anatomic features, as myocytes are less well-developed before 32 weeks of gestation, and the autonomic nervous system is considerably less active; 2) specific physiological features at extreme prematurity, when the ductus arteriosus is more likely to be patent with left-to-right or bidirectional shunt, ventricular distensibility is limited affecting preload, and the reserve for ventricular contractility is reduced, like during fetal life.

Currently, several gaps of knowledge complicate the generalizability of the research conducted on the field of neonatal hemodynamic that need to be addressed.

First, there is no validated score to diagnose circulatory insufficiency in this high-risk population. Blood pressure is a poor surrogate of systemic and organ blood flow during the transitional period from intra- to extra- uterine life [10–12]. This state is characterized by increased peripheral vascular resistance and therefore increased afterload, which may cause myocardial dysfunction and impaired blood flow in spite of ‘normal’ central blood pressure [5,13]. An alternative approach focuses on evidence of blood flow distribution and poor perfusion. We conducted the first pilot, randomized placebo-controlled trial (RCT) on dobutamine for early hemodynamic insufficiency, defined as low superior vena cava (SVC) flow in preterm infants below 31 completed weeks’ gestation [4]. Several routine parameters of circulatory impairment were prospectively collected. A retrospective analysis of the non-randomized infants (SVC flow within the first 24h from birth above 41 mL/kg/min) found SVC flow <51 mL/kg/min, mean arterial blood pressure (MBP) <gestational age (GA)-5 mmHg, and lactate above 4 mmol/L as the biomarkers that better predicted combined adverse outcome (death or severe intracranial hemorrhage or white matter damage) [3]. Therefore, the use of a combination of biomarkers will better define the population at risk of end-organ damage due to abnormal blood flow distribution that eventually would benefit of cardiovascular treatment.

Second, the best therapeutic approach to treat early hemodynamic insufficiency deserves further research. Dobutamine directly stimulates β-adrenergic receptors and is generally considered a selective β1-adrenergic agonist. In therapeutic doses, dobutamine also has mild β2 – and α1 – adrenergic receptor agonist effects, which are relatively balanced and result in minimal net direct effect on systemic vasculature [14–16]. Other sympathetic amines, such as dopamine or epinephrine, may exert predominant alpha-adrenergic effects, therefore increasing afterload and causing further impairment of myocardial performance and blood flow distribution to organs [5,12].

Several small observational studies and RCTs have reported cardiovascular effects of dobutamine in the newborn [15–23]. Only 2 RCT trials have used SVC flow as entry criteria and to monitor treatment response [4,24]. Osborn randomized 42 preterm infants below 30 weeks’ gestation with SVC flow <41 mL/kg/min to receive a normal saline bolus followed by dopamine or dobutamine, dose range 10–20 μg/k/min. At the highest dose reached, dobutamine produced significantly greater increases in SVC flow than dopamine [17]. Severe intraventricular hemorrhage was lower in dobutamine than in dopamine group (5% and 35%, respectively). Follow-up of 13 surviving infants at three years showed more disability and lower developmental quotients in dopamine treated infants but similar combined rates of death and disability were found [25]. A more recent pilot placebo-controlled trial on dobutamine for low SVC flow conducted by our group showed trends to improved clinical and biochemical parameters of hemodynamic insufficiency in infants treated with dobutamine [4]. The follow up of this cohort up to 6 years showed no differences in combined outcome (mortality or neurodevelopmental impairment) between intervention and control group [26].

The information gathered regarding dobutamine pharmacokinetic (PK) does not focus on the preterm infant [27–29]. Pellicer et al. [30] have recently reported on the elimination half-life of dobutamine in the target population, that is the preterm infant below 33 completed weeks’ gestation, which is crucial for the proposed study design. Elimination half-life is needed to calculate dobutamine steady state and, therefore, the time to measure the effect at any given dose. This study showed high inter-individual variability in the cardiovascular response to dobutamine administration in a homogeneous population [30].

The literature supports the further development of dobutamine as a treatment for early hemodynamic insufficiency in the preterm infant [17,22,31]. This study protocol aims to gather additional information on dobutamine PK and pharmacodynamic (PD) effects in the preterm newborn. We hypothesize that, in infants below 33 completed weeks’ gestation with transitional hemodynamic insufficiency, a continual reassessment method will allow to define the dose-range of dobutamine that most likely will have an effect on SVC flow, in other words, the optimal starting dose.

## Materials and methods

### Study design

The NeoCirc-002 trial is a multicenter, low-intervention clinical trial that will be conducted in three level-III Neonatal Intensive Care Units (NICUs) in Madrid, Spain: La Paz University Hospital, Quironsalud Madrid University Hospital, and 12 de Octubre Hospital.

### Study objectives and endpoints

#### Study objectives.

Primary study objective: to determine the minimum effective dose of dobutamine required to treat low SVC flow (<51 mL/kg/min) in infants below 33 completed weeks’ gestation (short- term pharmacodynamic (PD) objective).

Secondary study objectives: to assess the proportion of infants who maintain an acceptable hemodynamic status with the dobutamine infusion alone in the first 72h from birth (efficacy objective); to evaluate the safety of dobutamine for the whole study population (safety objective); to determine the variables that explain the interindividual PK/PD variability.

#### Study endpoints.

Primary endpoint: The minimum dobutamine dose to reach and maintain an SVC flow above 55 mL/kg/min on an echocardiogram performed at 1 and 3 hours after infusion of the allocated dose.

#### Secondary endpoints:

- The proportion of neonates achieving and maintaining a clinically acceptable hemodynamic status with the dobutamine infusion alone in the first 72 hours from birth. Acceptable hemodynamic status is defined as the achievement and maintenance of dose success during the first 72 h from birth. The loss of such acceptable hemodynamic status occurs whenever there is a change in therapeutic strategy that involves cardiovascular treatment other than dobutamine alone due to exceeded safety parameters, treatment failure of the investigational infusion or the need for rescue treatment or death. Patients with a loss of acceptable hemodynamic status in the first 72 h from birth, but with confirmation that the underlying etiology of circulatory failure is different from transitional circulation adaptation, will be excluded. These include sepsis, hemodynamically significant patent ductus arteriosus, air trapping (lung over distension), pneumothorax, and pleural or pericardial effusion.
- The absolute and relative frequencies of adverse events (AEs) and severe adverse events (SAEs), to be recorded and compared between groups.
- To determine the correlation between PK and PD

### Eligibility criteria

#### Inclusion criteria.

Infants are considered eligible for the study if born up to 32(+6) weeks’ gestation, suffer hemodynamic insufficiency, defined as SVC flow <51 mL/kg/min within the first 72h from birth, and signed and dated informed consent, prior informed assent (opt-out) or deferred consent is provided.

#### Exclusion criteria.

Infants meeting any of the following conditions will be excluded from participation in the trial: clinical decision not to provide life support, severe congenital hydrops fetalis, the infant is already on dobutamine treatment, congenital malformations likely to affect cardiovascular adaptation, chromosomal anomalies, or lack of parental signed informed consent.

### Intervention description

#### Investigational medicinal product (IMP) and dose assignment:.

The IMP will be commercial dobutamine (Dobutamine Generis, concentration 12.5 mg/mL) in a single, standard concentration. It will be impossible to blind caregivers or assessors to the dose used.

Once the patient has fulfilled the inclusion criteria, he or she will be allocated to a dosage regimen *(see section below, Statistical analysis).*

#### IMP administration.

IMP for infusion will be prepared and administered with strict standardization of the administration system to minimize systematic errors due to differences in infusion set-ups. This will ensure the precise time at which dobutamine enters the body can be recorded (t0).

The infant’s treatment will be guided by the attending physician who will use the study medication under the premise of keeping a minimum time of 60 minutes from the effective infusion of dobutamine (t0) for PD assessment. Once the primary PD variable has been assessed further management will be at the discretion of the attending physician. Therefore, if there is no PD response according to protocol with the allocated starting dose, or even if there were, the clinical situation of the patient so recommends it, the responsible physician will decide on further treatment (rescue treatment).

### Study assessments and procedures

**Fig 1:**
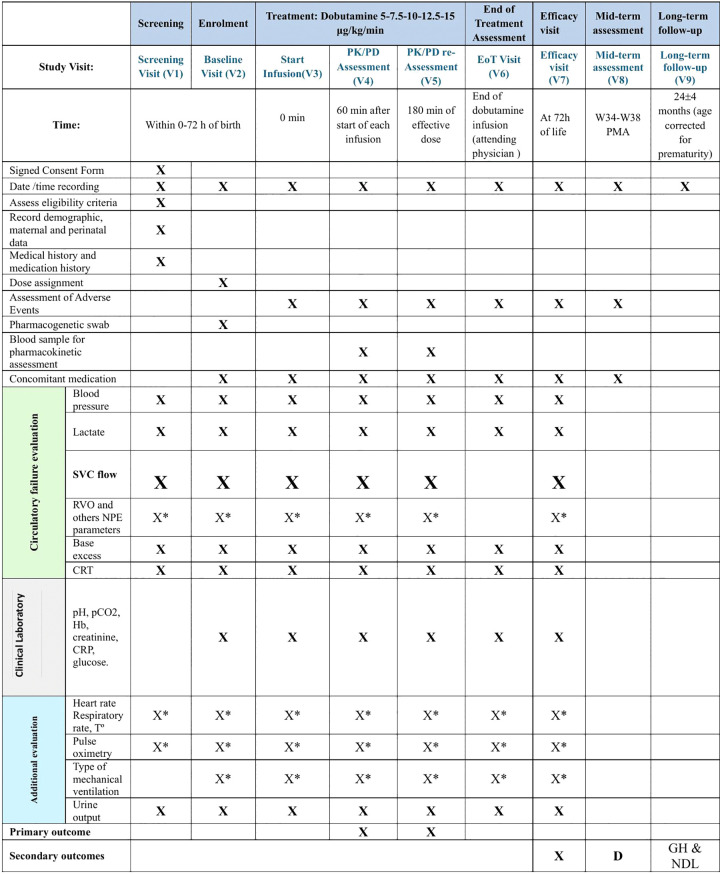
Schedule of assessments. RVO: right ventricular output; NPE: Neonatologist performed echocardiography; CRT: capillary refill time; Hb: hemoglobin; CRP: C-reactive protein; EoT: End of treatment. **X**: Indicates mandatory procedures that should be entered into the eCRF. X*: Indicates data entered into the eCRF if available. **D:** Main neonatal diagnoses: bronchopulmonary dysplasia, necrotizing enterocolitis, spontaneous bowel perforation, early infection, nosocomial infection, persistent ductus arteriosus, retinopathy of prematurity and cranial ultrasound diagnosis of severity of brain damage. **Neonatologist performed echocardiography (NPE) parameters:** Right ventricle output (mL/kg/min), left ventricular output (mL/kg/min), tricuspid annular plane excursion (mm), left ventricular ejection fraction (as Simpson, %) and patent ductus arteriosus: diameter (mm), ductal systolic velocity (m/sec), ductal diastolic velocity (m/sec), left pulmonary artery diastolic velocity (m/sec), descending aorta diastolic flow (forward, absent, reversed) and celiac trunk diastolic flow (forward, absent, reversed). **Type of mechanical ventilation:** non-invasive mechanical ventilation, conventional mechanical ventilation, high-frequency oscillatory ventilation, high-flow nasal cannula or none. **GH & NDL:** anthropometry (weight, length and head circumference), general health (admissions, medications, additional diagnoses), structured neurological exam, neurodevelopmental test.

The schedule and type of assessments to be conducted at each study visit are summarized in **Fig 1**.

The following study visits are to be accomplished:

A. Screening Visit (Time: within 0–72 hours of birth): Eligibility will be addressed and informed consent is obtained.
B. Enrolment: Baseline Visit (Time: within 0-72 hours of birth). The infant is enrolled into the trial. Pharmacogenetic study is accomplished (sample of oral mucosa is obtained), and dobutamine dose is assigned.
C. Treatment: Start of IMP Infusion (Time 0 min). Dobutamine infusion will start following a strict standardized procedure. Dobutamine infusion should be at the stopcock closest to the catheter entry. Before dobutamine is connected to the stopcock, the whole system (including bionector) will be fed with the dobutamine solution, and the pump will be switched on for a few minutes to ensure that no pulling out effect is added at start. The pump will be switched off and the system will be connected to the stopcock. Finally, the pump will be switched on and the time will be registered (t switch on).
D. Treatment: PD/PK Assessment (Time: 60 min after dobutamine dose infusion enters blood stream (t0)). The patient PD response to the dobutamine treatment should be evaluated at 60 minutes from the start of real dose infusion, to establish if the treatment success dose (SVC flow above 55 mL/kg/min) has been achieved. Blood sample (1 mL) will be taken for PK analyses. Real dose infusion is defined as the time (t0) when the study medication enters blood stream, which is calculated according to the following equation: t0= t switch on + td; td (min)= (volume (mL)/ velocity (mL/h)) x 60 (min/h), where volume refers to the catheter lumen plus the stopcock dead volume, and velocity refers to the summation of the infusion rate of all the infusions coming through the line.
E. Treatment: PD/ PK re-assessment Visit (Time: 180 min of effective dose). The patient PD response to dobutamine treatment should be re-evaluated at 180 minutes of dobutamine dose infusion that reached the PD target (SVC flow>55 mL/kg/min). A second one milliliter of blood sample will be obtained.
F. End of treatment. Weaning and stopping of the dobutamine infusion will be at the criteria of attending physician.
G. Efficacy Visit (Time: At 72 hours). To test whether dobutamine alone achieved and maintained an acceptable hemodynamic status during the first 72 h from birth (secondary objective). The infants’ condition at this time point will be: infant is alive, and: SVC flow > 55 mL/kg/min; MBP> GA; decreasing serum lactate levels; capillary refill time (CRT) <4s; urine output > 1 mL/kg/h; dobutamine alone was used (regardless the dose). Any other condition justifying treatment failure should be reported at this stage.
H. Mid-term follow up: at 36±2 weeks postmenstrual age (PMA) when the main neonatal diagnoses will be recorded, including worst cranial ultrasound (cUS) findings, based on the last cUS describing the most severe brain lesions.
I. Long-term follow-up: at 24 months corrected for prematurity when anthropometry, general health data, structured neurological exam, and neurodevelopmental test will be recorded.
J. End of study: when the patient has completed the study visits.

### Biological sample collection and analysis

For the PK assessment, 1-1.5 mL of blood will be collected 60 minutes after the start of the allocated dobutamine infusion and at 180 minutes of effective dobutamine dose infusion. A stabilizing solution containing ascorbic acid and citric acid will be added to each sample, which will then be maintained for at least 3 hours at 5 ± 5 °C prior to centrifugation. Samples will be centrifuged at 22 (±2)ºC and 3000g for 10 minutes to subsequently obtain a plasma sample (minimum volume: 160uL). These samples will be stored at −80 ± 10 °C until analysis.

Pharmacogenetic study of genetic polymorphisms and enzymatic activities will be performed to comprehensively characterize dobutamine treatment response in very preterm infants during transitional circulatory insufficiency [32–34]. The buccal swabs for pharmacogenetic analyses will be stored at −20 to −80ºC.

All biological samples will be sent in a single batch at the Central Research and Clinical Trials Unit (UCICEC) once the sample size is completed and enrolment is closed. UCICEC will forward the biological samples for PK and pharmacogenetic analyses to the corresponding labs.

### Discontinuation and withdrawal of participants

Discontinuation from commercial dobutamine does not mean discontinuation from the study, and remaining study procedures should be completed as indicated by the study protocol. If a clinically significant finding is identified after enrolment, the investigator or qualified designee will determine if any change in participant management is needed. Any new clinically relevant finding will be reported as an adverse event (AE).

A participant may be withdrawn from the study at any time at the request of his/her parent(s), or may be withdrawn at any time at the discretion of the investigator or sponsor for safety or administrative reasons.

If a participant withdraws from the study, he/she may request destruction of any samples taken and not tested, and the investigator must document this in the site study records.

The reason for participant discontinuation or withdrawal from the study will be recorded on the early discontinuation form of the Electronic Case Report Form (eCRF).

Subjects who sign the informed consent form and are assigned a dose but do not receive the study intervention will be excluded from the intention-to-treat population and may therefore be replaced. There will be no replacement of subjects who withdraw after they have been enrolled in the study and received the study drug.

### Plans to promote participant retention and complete follow-up

Lost to follow up is highly improbable unless early discharge/transfer to lower care unit (before 34 weeks) which is rather infrequent. In these cases, the research team are committed to contact the families/transfer hospital for a required study visit. Should the participant continue to be unreachable, he/she will be considered to have withdrawn from the study with a primary reason of lost to follow-up.

### Concomitant care and rescue treatments

Overall care of the infant will follow the policies currently in use in the unit. There is no specified ‘per-protocol’ concomitant medication or treatment. Other important co-interventions will follow specific center protocols and will be recorded in the eCRF. All allowed medications that have been started before screening, and those prescribed for treatment of emerging illnesses that arise after screening will be allowed and documented in the eCRF. Precautionary medications, due to their effects on dobutamine metabolism or PD include entacapone and beta-blockers. Alternative fluid bolus or vasopressor-inotrope medications will be used only as rescue therapy.

### Rescue treatment

A change in treatment strategy using rescue treatments is allowed if no clinical response to the study intervention is considered by the attending physician, when: MBP < GA-5 mmHg; and one or more of the following: SVC flow < 51 mL/kg/min (after 1 hour), rising serum lactate, base excess being more negative, or CRT ≥ 4sec.

Rescue treatments include: additional fluid bolus; vasopressor-inotrope therapy, apart from commercial dobutamine; corticosteroids; or other treatment administered to treat hemodynamic insufficiency.

When rescue treatment is needed physicians will be encouraged to consider dobutamine up-titration option first, to try to maintain the infant in the IMP only treatment corridor as long as possible, if it is the best option for the infant.

### Statistical analysis

#### Sample size, allocation to dose regimens and statistical methods for primary outcome.

To calculate the dose-escalated sample size, the Bayesian Method of Continual Reassessment [35–39] will be used, using the following a priori probability of success for each dose:

a. 5 mcg/kg/min → 45–50%
b. 7.5 mcg/kg/min → 70–75%
c. 10 mcg/kg/min → 80–85%
d. 12.5 mcg/kg/min → 90–95%
e. 15 mcg/kg/min → 95–100%

In addition, a target probability of 80%, maximum sample size of 30 patients and a confidence level of 90% will be considered.

Stopping rules: the planned number of 30 subjects is reached; the estimated efficacy is too low for all dose levels; suitable estimation of the minimum effective dose is obtained, based on the predictive gains (mean and maximum) of further patients inclusions on the response probability and on the width of its credibility interval lower than 5%.

Statistical analyses will be performed using software RStudio (version 4.1.1) and the web application of the Division of Translational Research and Applied Statistics, University of Virginia.

The estimated number of patients per dose is shown in **Table 1**:

**Table 1:** Treatment allocation scheme.

	STEP 1	STEP 2	STEP 3	STEP 4	STEP 5
**DOSAGE (mcg/kg/min)**	5	7.5	10	12.5	15
**EFFICACY (%)**	45	70	80	90	95
**PATIENTS (n)**	5	9	12	4	0

Once the patient has fulfilled the inclusion criteria, he or she will be allocated to a dosage regimen. The statistician will be informed about the therapeutic response observed for re-estimation of the posterior probability of success after each cohort of patients. The choice of allocation will be decided according to dose with the posterior probability closer to the target probability (80%).

The primary outcome will be evaluated by intention-to-treat analysis. In case of missing data, the multiple imputation statistical method will be used. No interim or subgroup analyses are planned.

### Statistical methods for secondary outcomes

#### Safety endpoint.

Safety analysis will be conducted for the whole study population as well as for the five treatment groups separately. AEs and SAEs will be reported with absolute and relative frequencies per treatment group along with the corresponding 95% CIs (two-sided). Differences between different groups (each of the dose regimens separately) will be compared using Chi-squared Test. For all comparisons 95% CIs (two-sided) will be provided.

#### PK and pharmacogenetic analysis.

PK analysis will be performed for the complete study population. Differences between different groups and patients will be compared using Chi-squared Test. For all comparisons 95% CIs (two-sided) will be required.

### Consent procedures

By definition, this is a trial conducted on a vulnerable population, both from the perspective of the subject but also from the point of view of the parents. For this reason, a strategy for recruitment is in place to avoid overloading parents with information during the critical period after birth when they are more vulnerable: information about the trial is public by leaflets; whenever possible parents will give written informed consent antenatally, after explanation of the aims, methods, benefits and potential hazards of the trial; finally, parents that have not yet given written informed consent for the trial will be approached as soon as possible after their baby’s birth and provided with the relevant parental information.

Given that the condition can be considered as an emergency in some situations, and treatment should be indicated if circulatory failure is present regardless of the clinical trial, deferred informed consent and prior informed assent (opt-out with enrollment as default) would be applicable [Article 35 of the Regulation of the European Union on Clinical Trials (Regulations (EU) no536/2014)].

The investigator will certify that is not aware of any objections to participate in the clinical trial previously expressed by the legally designated representative. Informed consent shall be sought to continue the participation of the subject in the clinical trial, and information on the clinical trial shall be given to the parent or the legally designated representative.

### Additional consent provisions for collection and use of participant data and biological specimens

The informed consent will also include aspects related to biological samples handling for pharmacokinetic/pharmacodynamic (PK/PD) and pharmacogenetic analyses.

### Adverse event reporting and harms

All AEs that occur during the trial will be documented in the patient’s medical history. AEs that were related with the IMP or not related but serious will be recorded: description, start and end date, severity, seriousness, evaluation in relation to the investigation drug, actions taken and outcome.

Serious Adverse Reaction (SAR) that occur during the trial must be notified to the sponsor within a maximum period of 24 hours from the time of knowing about the event. Also, the investigator shall complete and sign the SAR report form and send it by fax or email to UCICEC. The sponsor or whoever assumes the tasks delegated by the sponsor shall keep detailed records of all the SARs notified by the investigators.

### Data management

Biomedical Research Foundation of La Paz University Hospital will be responsible for the data management and storage of the study data (on a database) including data storage and backup, data validation, and data coding.

After conducting all data validation and the final review, the study database will be considered as completed and its containing data as reliable. At this moment, the study database will be closed and transferred to the Biostatisticians team for data analyses. At the end of the study, a copy of the site-specific records will be provided to principal investigator.

### Confidentiality

The study staff will ensure that the participant´s anonymity is maintained. The participants will be identified only by a participant code on the eCRF and any electronic database. All documents will be stored securely and only accessible by study staff and authorized personnel.

Applicable regulations for storage, transmittal and disclosure of patient information will be always followed. The study will comply with the Data Protection Legislation.

Following formal admission to the study, patient data will be recorded in the hospital case record in the usual way including the circumstances of their entry to the study. Additionally, data will be held in eCRF. These files will be identified by a study code, date of birth and participant code only.

Results of the study may be communicated at scientific meetings and will contribute to the scientific literature. At no time, will this be done in such a way that an individual patient may be identified.

### Data monitoring committee

The Data Monitoring Committee (DMC) will operate under the standard procedures of the Spanish Clinical Research Network (SCReN). SCReN, an established network supporting independent and academic clinical research, will oversee the monitoring process. It will be composed of independent experts in clinical trials and will be responsible for overseeing patient safety, reviewing accumulating trial data, and ensuring the scientific validity of the study.

### Ethics approval and consent to participate

The study was approved by the local Ethics Committee (Ethics Committee of La Paz University Hospital, internal code: 2023.270) and by the Spanish Agency of Medicines and Medical Devices (AEMPS). Informed consent from parents or legal representatives of patients is mandatory for study participation, adhering to the protocol guidelines.

### Protocol amendments

All significant protocol modifications will be promptly communicated to all relevant parties. This includes investigators, Institutional Review Boards (IRBs), and trial registries. Non-substantial amendments will be recorded and filed.

### Dissemination plans

The results of the trial will be considered for publication in peer-reviewed journals and presentation at scientific symposia or conferences.

### Trial status

This manuscript adheres to the trial protocol version 2.0, dated November 2023. Recruitment of participants began on June 1, 2024, and is currently ongoing. Recruitment is anticipated to be completed by June 1, 2026, data collection by June 1, 2028, and the final results are expected to be analyzed and reported by late 2028.

## Discussion

This multicenter, dose-finding trial on dobutamine, conducted in a population of very preterm infants who suffer early hemodynamic insufficiency during transitional circulation, aims to define the minimum dose of dobutamine that has a substantial positive change on the baby’s hemodynamic condition, defined by SVC flow. NeoCirc-002 has been proposed in the light of the results of previous research of our group on the use of cardiovascular support, mainly catecholamines, to treat early hemodynamic insufficiency in the very preterm infant [3,4,10,11,26,30]. Dobutamine is a well-known inotrope widely used in a vulnerable population, such are neonates. Dobutamine has been included in the priority list of studies into off-patent pediatric medicinal products revised in 2010 by the European Medicines Agency (EMA). Age-related differences in absorption or metabolism result in suboptimal treatments. The lack of formal studies in this population results in insufficient data on safety. This is essential as neonates, especially preterm neonates, and adults differ in physiological capabilities, pharmacokinetic profile and pharmacodynamic characteristics.

This study will use as PD biomarker for treatment response the systematic assessment of SVC flow by echocardiography, reported as a relevant parameter of circulatory impairment occurring shortly after birth [3,4,24,40]. In a context where a validated scoring system of circulatory failure is lacking, clinical staff now use dobutamine to treat low SVC flow in neonates and have observed trends towards improved long-term outcomes [25]. The fact that this is a multicenter trial adds value in supporting the viability of this approach.

This small study is absolutely unpowered to address key issues regarding the efficacy and safety of the proposed intervention, dobutamine treatment for low SVC flow during transitional circulation, that should be answered with appropriate study designs against relevant clinical outcomes. However, the proposed study design chases to answer a key question in the planning of future randomized clinical trials in this topic, with minimal number of study participants. As the dose of dobutamine most likely to have an effect on SVC in this age group and the components of the inter-individual variability are currently unknown, it is paramount that research is carried out to find the optimal dose.

## Supporting information

S1 File SPIRIT checklist. (PDF)

S2 File Study protocol. (PDF)
