# Supplementary material for: Dose-finding for dobutamine during transitional circulation in the very preterm infant: The study protocol
Source: PLoS One. 2025 Dec 19;20(12):e0338307. doi: 10.1371/journal.pone.0338307 (PMC12716709; doi:10.1371/journal.pone.0338307)
Supplement: S2 File — (PDF) [file pone.0338307.s002.pdf]

Dose-finding for dobutamine during transitional circulation in the very preterm infant

NeoCirc-002 TRIAL

Chief Investigator: Adelina Pellicer

Collaborator investigator: Patricia Álvarez García

María Sánchez Holgado

María Carmen Bravo

Sponsor: Fundación para la Investigación Biomédica

Hospital Universitario La Paz (FIBHULP)

Funded by:

Version number: Version v. 2.0. (includes non-substantial modification number 1 28/05/2024)

Date: 17/11/2023

EU CT Number: 2023-504915-34-00

STATEMENT OF COMPLIANCE

Sponsor will ensure that this study is conducted in accordance with the protocol, the principles of the Declaration of Helsinki, International Conference on Harmonisation Good Clinical Practice (ICH GCP) and in full conformity with relevant regulations.

The protocol, informed consent form, patient's legal representative/parent's information sheet and any applicable documents will be submitted to the appropriate Ethics Committee (EC), Regulatory Authority and any other Regulatory Body for written approval according to applicable regulations. Approval by regulatory bodies of both the protocol and the consent form must be obtained before any participant is enrolled.

All substantial amendments to the original approved documents will be also sent to an appropriate EC, Regulatory Authority and any other Regulatory Body for written approval according to applicable regulations; a determination will be made regarding whether a new consent needs to be obtained from participants who provided consent, using a previously approved consent form.

The Principal Investigator will assure that no deviation from, or changes to the protocol will take place without prior agreement from trial sponsor, funding agency, and documented approval from the appropriate EC, except where necessary to eliminate an immediate hazard(s) to the trial participants. All personnel involved in the conduct of this study have completed Human Subjects Protection and ICH GCP Training.

The Sponsor certifies that the possible risks of the study are by the civil liability insurance that the Madrid Health Service (SERMAS) has contracted.

1 PROTOCOL SUMMARY

1.1 SYNOPSIS

**Title:**	Dose-finding for dobutamine during transitional circulation in very preterm infants
**Study Description:**	Single centre, dose finding trial to establish the minimum effective dose of dobutamine required to treat haemodynamic insufficiency, defined as low superior vena cava (SVC) flow, in infants below 33 weeks' gestation during transitional circulation (first 72 hours from birth).<br>It is a low-intervention clinical trial.
**Objectives:**	Primary objectives:<br>• To determine the minimum effective dose of dobutamine required to treat low SVC flow (<51 ml/k/min) in very preterm infants **(short- term pharmacodynamic (PD) objective).**<br>Secondary objectives:<br>• To assess the proportion of infants who maintain an acceptable haemodynamic status with the dobutamine infusion alone in the first 72h from birth (efficacy)<br>• To evaluate the safety of dobutamine for the whole study population as well as for the seven treatment groups separately.<br>• To determine the individual variables that explain the interindividual pharmacokinetic (PK)/PD variability.
**Endpoints:**	Primary endpoints:<br>• **Short-term PD endpoint:** Minimum dobutamine dose to reach and maintain an SVC flow above 55 ml/k/min on an echocardiogram performed at 1 and 3 hours after effective infusion of the allocated dose [A].<br>Secondary endpoints:<br>• Proportion of neonates achieving and maintaining a clinically acceptable haemodynamic status [B] with the dobutamine infusion alone in the first 72 hours from birth.<br>• Absolute and relative frequencies of adverse events (AEs) and severe adverse events (SAEs), to be recorded and compared between groups.<br>• To determine the correlation between PK and PD.<br>[A] The effective start of the infusion (t0) will be calculated as the time at which the infusion pump is switched on plus the empirical value for the interval arising from the dead space. We summarize t0 as "the time at which dobutamine is expected to reach the circulation"

	[B] Acceptable hemodynamic status is defined, for the purposes of this study, as the achievement and maintenance of dose success during the first 72 h from birth. The loss of such acceptable haemodynamic status occurs whenever there is a change in therapeutic strategy that involves cardiovascular treatment other than dobutamine alone due to exceeded safety parameters, treatment failure of the investigational infusion or the need for rescue treatment or death; any additional fluid bolus is considered as cardiovascular treatment.
**Study Population:**	Preterm neonates of gestational age (GA) less than 33 weeks with a postnatal age below 72 hours.<br>**Inclusion criteria**: GA ≤ 32(+6) weeks and early haemodynamic insufficiency defined as SVC flow <51 ml/kg/min in the first 72h after birth.
**Phase:**	I-II
**Description of Sites/Facilities:**	Level 3 C Neonatal Intensive Care Unit (NICU) in Madrid, Spain.
**Description of study Intervention:**	Study medication: commercial dobutamine (Dobutamine Generis®, concentration 12.5 mg/ml). Route of administration: Intravenous.<br>Blinding: a single standard concentration will be available. It will be impossible to blind carers or assessors to the dose used.
**Dose regimens:**	A. 5 microg/kg/min<br>B. 7.5 microg/kg/min<br>C. 10 microg/kg/min<br>D. 12.5 microg/kg/min<br>E. 15 microg/kg/min.
**Allocation to dose regimens:**	Participants will be allocated to a dosage regimen by the study statistician.<br>The statistician will be informed about the therapeutic response observed for re-estimation of the posterior probability of success after each cohort of 3 patients. The choice of allocation will be decided according to dose with the posterior probability closer to the target probability (80 %).
**Stopping rules**	1. the planned number of 30 subjects is reached;<br>2. estimated efficacy is too low for all dose levels;<br>3. suitable estimation of the first dose is obtained, based on the predictive gains (mean and maximum) of further patients' inclusions on the response probability and on the width of its credibility interval lower than 5%.

<table>
<tr><td>PK assessments</td><td>Samples for dobutamine will be taken at 60 min and 180 min after the allocated dose had time to enter the systemic circulation. In the case the targeted PD effect is reached with a different dose, the second PK sample will be withdrawn 180 min after effective dose infusion.</td></tr>
<tr><td>Study Duration:</td><td>Start date is the first quarter of 2024. The study is open and patients' enrolment starts and continues along 12-months period. The estimated time until completion of data analyses is of 4 years.</td></tr>
<tr><td>Participant Duration:</td><td>Participant will be observed at PMA 36 (2) weeks for main neonatal outcomes assessment and long-term follow-up at 24 (±4) months of age corrected for prematurity.</td></tr>
<tr><td>Statistical analysis</td><td>To calculate the dose-escalated sample size, the Bayesian Method of Continual Reassessment was used, using the following a priori probability of success for each dose:
A. 5 mcg/kg/min → 45-50%
B. 7.5 mcg/kg/min → 70-75%
C. 10 mcg/kg/min → 80-85%
D. 12.5 mcg/kg/min → 90-95%
E. 15 mcg/kg/min → 95-100%

In addition, a target probability of 80%, maximum sample size of 30 patients and a confidence level of 90% was considered. The statistical software RStudio (version 4.1.1) and the web application of the Division of Translational Research and Applied Statistics, University of Virginia, were used.

Sample size: the estimated number of patients per dose is shown below:
<table>
<tr><th></th><th>STEP 1</th><th>STEP 2</th><th>STEP 3</th><th>STEP 4</th><th>STEP 5</th></tr>
<tr><td>DOSAGE
(mcg/kg/min)</td><td>5</td><td>7.5</td><td>10</td><td>12.5</td><td>15</td></tr>
<tr><td>EFFICACY (%)</td><td>45</td><td>70</td><td>80</td><td>90</td><td>95</td></tr>
<tr><td>PATIENTS (n)</td><td>5</td><td>9</td><td>12</td><td>4</td><td>0</td></tr>
</table>

After the simulation and at each dose-escalated, an implementation of the results obtained will be performed, recalculating the efficacy estimates per dose and the required number of patients. This escalation will stop when the expected efficacy is achieved.</td></tr>
</table>

1.2 Schema

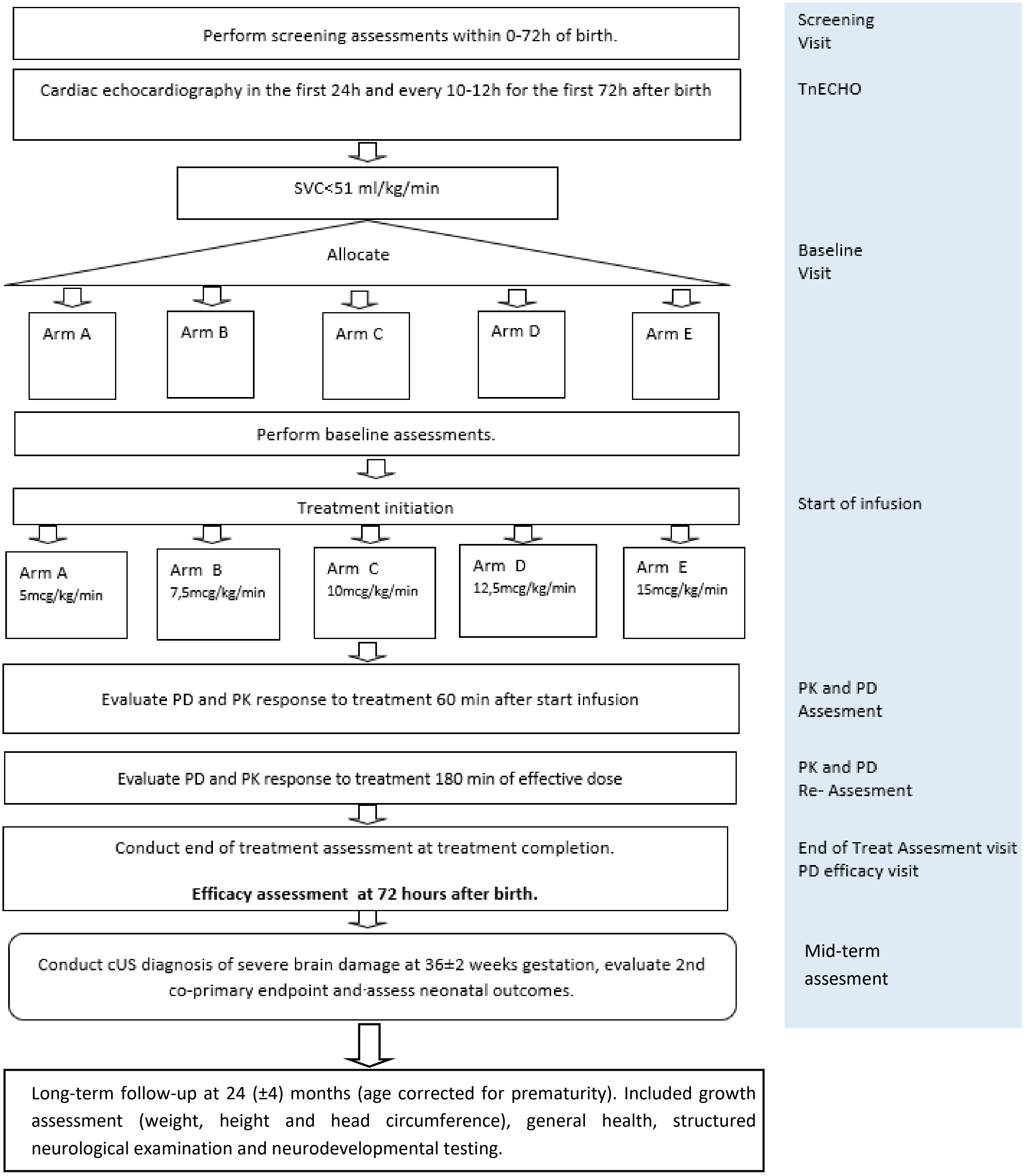
Perform screening assessments within 0-72h of birth.
Cardiac echocardiography in the first 24h and every 10-12h for the first 72h after birth
SVC<51 ml/kg/min
Allocate
Arm A
Arm B
Arm C
Arm D
Arm E
Perform baseline assessments.
Treatment initiation
Arm A 5mcg/kg/min
Arm B 7,5mcg/kg/min
Arm C 10mcg/kg/min
Arm D 12,5mcg/kg/min
Arm E 15mcg/kg/min
Evaluate PD and PK response to treatment 60 min after start infusion
Evaluate PD and PK response to treatment 180 min of effective dose
Conduct end of treatment assessment at treatment completion.
Efficacy assessment at 72 hours after birth.
Conduct cUS diagnosis of severe brain damage at 36±2 weeks gestation, evaluate 2nd co-primary endpoint and assess neonatal outcomes.
Long-term follow-up at 24 (±4) months (age corrected for prematurity). Included growth assessment (weight, height and head circumference), general health, structured neurological examination and neurodevelopmental testing.
Screening Visit
TnECHO
Baseline Visit
Start of infusion
PK and PD Assesment
PK and PD Re- Assesment
End of Treat Assesment visit
PD efficacy visit
Mid-term assesment

1.2 SCHEDULE OF ASSESSMENTS(SoA)

		Screening	Enrolment	Treatment: Dobutamine 5-7.5-10-12.5-15 µg/kg/min			End of Treatment Assessment	Efficacy visit	Mid-term assessment	Long-term follow-up
**Study Visit:**		Screening Visit (a)	Baseline Visit (b)	Start Infusion	PK/PD Assessment	PK/PD re-Assessment	EoT Visit			
**Time:**		Within 0-72 h of birth		0 min	60 min after start of each infusion	180 min of effective dose	At treatment completion	At 72h	W34-W38 PMA	24±4 months (age corrected for prematurity)
Signed Consent Form		X								
Date /time recording		X	X	X	X	X	X	X	X	X**
Assess eligibility criteria		X								
Record demographic, maternal and perinatal data		X								
Dose regimen assignment			X							
Assessment of Adverse Events				X	X	X	X	X		
**Circulatory failure evaluation**	BP (Mean, Syst. Diast.)	X*	X	X	X	X	X	X		
	Lactate	X*	X*	X*	X*	X*	X*	X*		
	**SVC flow** and relevant parameters	X	X	X	X	X		X		
	RVO	X*	X*	Pd*	Pd*	Pd*		X		
	Base excess	X*	X*	Pd*	Pd*	Pd*	X	X		
	CRT	X*	X	Pd	Pd	Pd	X	X		
**Clinical Laboratory**	pH, pCO2, Hb, creatinine, CRP, glucose.		X*	X*	X*	X*	X*	X*		
**Additional evaluation**	Heart rate	X*	X	X*	X	X*	X*	X		
	Pulse oximetry	X*	X	X*	X	X*	X*	X		
	Type of mechanical ventilation		X	X	X	X	X	X		
	Urine output	X	X	X	X	X	X	X		
**primary outcome**					X			X		
**Secondary outcomes**									D	

X : Indicates Mandatory Procedures that should be entered into the eCRF;

X*: Indicates data entered into the eCRF if available;

Pd*: Indicates non-mandatory pharmacodynamics assessment at the visit that should be entered into the eCRF if available

D: Included bronchopulmonary dysplasia, necrotizing enterocolitis, retinopathy of prematurity and cUS diagnosis of severity of brain damage (cerebral haemorrhage grade II or III, periventricular haemorrhagic infarction, white matter injury, post-haemorrhagic ventricular dilatation, cerebellar haemorrhage, or cerebral atrophy).

a) Infants are screened for inclusion into the study within 0-72 hrs of birth. Screening Visit finishes when infant is enrolled and allocated (Baseline Visit). Sign informed consent form prior to any assessment in the trial and record date of signing the informed consent form; except in the case of deferred consent.

b) Infants that fulfil inclusion criteria are allocated to intervention; provided there are no exclusion criteria according to protocol.

X**: Growth assessment (weight, height and head circumference), general health, structured neurological examination and neurodevelopmental testing included.

2 INTRODUCTION

2.1 STUDY RATIONALE

2.1.1 The Condition to Address and Current Limitations of Knowledge

Haemodynamic insufficiency occurs when there is a state of poor organ blood flow resulting in cellular energy failure due to an inability of tissue oxygen delivery to satisfy tissue oxygen demand. Haemodynamic insufficiency after birth is commonly seen in babies born prematurely. The condition has a significant clinical impact. A final common pathway is seen but the consequences of functional impairment are diverse. During the neonatal period haemodynamic insufficiency can lead to loss of existing function and the loss of function that is yet to be developed.

The first hours after birth differ markedly from all other periods in human life and are characterised by a unique and dynamically changing anatomy. This is accompanied by striking changes in function as the circulatory system of the healthy newborn adapts rapidly to life outside the womb. Haemodynamic physiology and pathophysiology changes are specific to this age-group and generate unique clinical challenges.

In current clinical practice, diagnostic and therapeutic approaches are different when managing neonates born at less than 32 weeks gestation compared to older infants. Epidemiologic studies indicate an association between haemodynamic abnormalities in the days after birth in the preterm infant and adverse outcomes (5-11).

There are anatomic features of the cardiovascular system that render neonates born at less than 32 weeks' gestation unique. These relate to:

- The myocardium (myocytes are less well-developed before 32 weeks of gestation);
- Neuronal control (the autonomic nervous system is considerably less active before 32 weeks of gestation).

The specific physiological features of the adaptation to birth at extreme prematurity are:

- the ductus arteriosus (this is more likely to be patent with left-to-right or bidirectional shunt among extremely preterm neonates than in more mature neonates);
- the extent of ventricular distensibility (affecting preload) which is less following birth before 32 weeks than following birth after 32 weeks gestation;
- the reduced reserve for ventricular contractility that is seen during foetal life.

A significant proportion of babies born at less than 32 weeks' gestation require cardiovascular treatment in the hours after birth, even in the absence of identifiable pathologies. This indicates that haemodynamic insufficiency in this age group can be related to an immature adaptation to birth.

The conventional approach to infants at risk of haemodynamic insufficiency is to monitor the babies using clinical signs, mostly blood pressure. However, blood pressure is a poor surrogate of systemic and organ (including brain) blood flow during the transitional period from intra- to extra- uterine life. This state is characterised by increased peripheral vascular resistance and therefore increased afterload, which may cause myocardial dysfunction and impaired blood flow in spite of 'normal' central blood pressure.

We propose an alternative approach, which focuses on evidence of blood flow distribution and poor perfusion. One pilot, randomised placebo-controlled trial on dobutamine for early haemodynamic insufficiency, defined as low SVC flow (<41 ml/kg/min), conducted in infants below 31 completed weeks' gestation, has been recently published (11). Eligible infants were prospectively evaluated for several parameters of circulatory impairment from birth, and randomised to dobutamine or placebo in case of low SVC flow within the first 24 hours. An analysis in retrospect of the non-randomised infants, looking at biomarkers that would qualify as a criterion for inclusion of patients in a future phase III clinical trial to demonstrate efficacy for cardiovascular treatment in the indication under investigation, found SVC flow <51ml/kg/min, mean arterial blood pressure (MABP)< gestational age (GA)-5 mmHg, and lactate above 4mmol/L as the biomarkers that better predicted combined adverse outcome (death or severe intracranial haemorrhage or white matter damage)(12). The use of a combination of biomarkers that have been proven to be as good predictors for adverse outcome, will better define the population at risk of end-organ damage due to abnormal blood flow distribution that eventually would benefit of cardiovascular treatment.

2.1.2 Why Dobutamine to Treat Early Haemodynamic Insufficiency in the Preterm?

Dobutamine increases myocardial contractility with no effect or negative effect on the peripheral vascular resistance (no change or decreased afterload). Taking into account the pathophysiology of early haemodynamic insufficiency explained above, these effects point to dobutamine as a potential first line inotrope for that condition in this vulnerable population. Other sympathetic amines currently used for haemodynamic support in the preterm infant, such as dopamine or epinephrine, may exert predominant alpha-adrenergic effects, therefore increasing afterload and causing further impairment of myocardial performance and blood flow distribution to organs.

We hypothesize that dobutamine would fit the needs in case of failure to adapt to transitional circulation shortly after birth in the preterm infant, born at 32(+6) weeks gestation or below, by improving cardiac performance without raising peripheral vascular resistance and consequently, preventing or limiting disturbances in organ blood flow distribution and tissue oxygen delivery.

2.2 BACKGROUND

2.2.1 The Study Agent

Dobutamine is a synthetic sympathomimetic amine that was originally developed for the treatment of congestive heart failure. Dobutamine contains an asymmetric carbon atom and two enantiomers: the (-)- and (+)- isomers of dobutamine. Dobutamine directly stimulates β-adrenergic receptors and is generally considered a selective β1-adrenergic agonist. In therapeutic doses, dobutamine also has mild β2 - and α1 - adrenergic receptor agonist effects, which are relatively balanced and result in minimal net direct effect on systemic vasculature. Dobutamine does not cause release of endogenous norepinephrine. The main effect of therapeutic doses of dobutamine is cardiac stimulation. While the positive inotropic effect of the drug on the myocardium appears to be mediated principally via β1-adrenergic stimulation, experimental evidence suggests that α1-adrenergic stimulation may also be involved and that the α1-adrenergic activity results mainly from the (-) -stereoisomer of the drug (14).

The dobutamine-induced cardiovascular response is influenced by developmentally regulated expression of adrenergic receptors, down-regulation of these receptors in relation to disease status, developmental differences in maturity of myocardium, and local production of vasodilators, such as endogenous nitric oxide and prostacyclin, among others.

2.2.2 Summary of Clinical Research on Dobutamine in the Target Population

In the newborn, several small observational studies have reported cardiovascular effects of dobutamine. Briefly, dobutamine showed improved left ventricular function, increased cardiac output (17-19), and a positive effect on blood flow at the cerebral, splanchnic and renal circulations (19). Whilst no studies have invasively examined the effects of dobutamine on pulmonary vascular pressure, some studies found that fraction of inspired oxygen decreased by an average of 8% in response to dobutamine infusion up to 20 μg/kg/min (19-21).

Randomised clinical trials (RCTs) conducted in preterm infants support the superiority of dopamine compared to dobutamine at increasing blood pressure (22-27). From a physiological perspective dobutamine would be expected to improve end-organ blood flow without necessarily affecting blood pressure. Accordingly, investigators have conducted studies to assess the effect of dobutamine on other aspects of haemodynamic insufficiency. So far, two therapeutic exploratory trials with dobutamine have been conducted (11) relating to the treatment of low superior vena cava (SVC) flow, used as an estimator of systemic blood flow (29). Osborn randomised 42 preterm infants below 30 weeks' gestation with low SVC flow (<41 ml/kg/min) to receive a normal saline bolus followed by dopamine or dobutamine, dose range 10 to 20 μg/k/min. At the highest dose reached, dobutamine produced significantly greater increases in SVC flow than dopamine (28). Severe intraventricular haemorrhage was lower in dobutamine than in dopamine group (5% and 35%, respectively). Follow-up of 13 surviving infants at three years showed more disability and lower developmental quotients in dopamine treated infants (30). However, these figures need to be taken with caution as more infants died in dobutamine group (similar combined rates of death and disability). A more recent pilot placebo-controlled trial on dobutamine for low SVC flow showed trends to improved clinical and biochemical parameters of haemodynamic insufficiency in infants treated with dobutamine (11).

The information regarding dobutamine pharmacokinetics (PK) in the preterm infant is scarce. Several studies evidenced the PK of dobutamine in children is linear within the investigated

infusion rates (0.5 to 20 µg/kg/min) (18, 31-33). In 27 children in intensive care with age ranging between 1 month and 16.6 years, the pharmacokinetics of dobutamine was found to follow a 1 or a 2-compartment model and was characterized by a considerable inter-individual variability: for an administration rate of 5 µg/kg/min, steady-state concentrations ranged between 3.79 and 400 ng/ml (34). Age, body weight, and sex were not related to the value of the PK parameters. In neonates, no association was found between plasma clearance and birth weight or gestational age (18).

Dobutamine was found to obey a threshold model, which consists in a lack of response when dobutamine concentration is below a threshold value, and, beyond this threshold, to a linear increase in the response for logarithmic increases in plasma dobutamine concentration (18, 31-33). However, the inter-individual variability in the cardiovascular response to dobutamine administration is high. Several studies have shown that different genetic polymorphisms may be related to this inter-individual variability, determining success or failure of circulatory therapy (50-52). We aim to analyse the pharmacogenetics of GNAS1 and β1-adrenoceptor polymorphism and different enzymes: COMT, MAO-A, MAO-B, because they are the main metabolizer enzymes of dobutamine.

2.2.3 The Importance of the Study

Dobutamine is a well-known inotrope widely used in neonates and children that suffer haemodynamic insufficiency. Dobutamine has been included in the priority list of studies into off-patent paediatric medicinal products revised in 2010 by the European Medicines Agency (EMA). Age-related differences in absorption or metabolism result in suboptimal treatments. The lack of formal studies in this population results in insufficient data on safety. This is essential as children, especially neonates, and adults differ in physiological capabilities, pharmacokinetic profile and pharmacodynamic characteristics. Their metabolic pathways, organic functions and metabolic rates, differ widely. Disparities also exist in terms of receptor functions, effector systems and homeostatic mechanisms. In addition, age, growth and development influence side effects, and the dose of medications is dependent on body weight or surface area.

The literature supports the further development of dobutamine as a treatment for early haemodynamic insufficiency in the preterm infant. Information regarding efficacy and safety of dobutamine in neonates, particularly in the preterm infant, is limited to small observational studies or RCTs that were not powered for the long-term outcomes and used fixed dosages selected to maintain blinding of measurement. Therefore, there is need for data on efficacy and safety.

In addition, it is important to note that a common clinical scoring system to evaluate haemodynamic insufficiency and poor tissue perfusion has not been developed.

The combined use of different techniques capable of monitoring the most important components of cardiovascular functions, such as echocardiography-derived parameters, cerebral oximetry, blood lactate, blood pressure or capillary refill time, are likely to provide more plausible information necessary for early recognition of impaired tissue perfusion in neonates. However, the components of the assessment, their thresholds and weighting need to be defined.

Echocardiographic measurements of superior vena cava (SVC) flow have been proposed as being a relevant marker of circulatory impairment occurring shortly after birth (11-12,29,43).

Clinical staff now use dobutamine to treat low SVC flow in neonates and have observed trends towards improved long-term outcomes (8,30).

NeoCirc-002 has been proposed in the light of the results of the pilot study (NeoCirc-001) (35) and the studies conducted by Bravo et al in a cohort of 127 preterm infants below 31 completed weeks (11, 12), that have answered key questions about the design of a randomized, phase III confirmatory trial on the efficacy and safety of dobutamine to treat early haemodynamic insufficiency in the preterm infant. As the dose most likely to have an effect on SVC in this age group is currently unknown, it is paramount that research is carried out to find the optimal dose.

2.4 RISK/BENEFIT ASSESSMENT

2.4.1 Known Potential Risks

Safety data on dobutamine use in neonates have not been systematically evaluated in RCTs, either in preterm or term newborns. Reported side effects of dobutamine in small RCTs conducted in preterm infants support the notion that it is well tolerated (11, 22-28).

RCTs comparing dopamine and dobutamine to treat systemic hypotension or low SVC flow found no differences on percentage change in heart rate (8, 36); however, cardiac arrhythmia was not systematically evaluated (36). Dobutamine-induced increase in heart rate may limit the use of high doses in some neonates. In addition, drug-induced decrease in the compliance of the myocardium as a result of significant increase in the myocardial tone may result from dobutamine use. Dopamine, but not dobutamine, has been shown to cause transient (reversible) reduction in serum levels of thyroid stimulating hormone, thyroxine and prolactin (27). No differences on short-term neonatal outcomes (mortality, structural brain damage, necrotizing enterocolitis, retinopathy of prematurity or chronic lung disease) were found, although follow-up of these variables is not complete in all trials. However, in a therapeutic exploratory trial conducted in preterm infants below 30 weeks' gestation who had low SVC flow in the first 12 h, the mortality rate in the dobutamine group was higher than the dopamine group but the difference was not statistically significant (28, 30).

Very recently an apoptotic effect of dopamine and dobutamine on neutrophils of 15 preterm infants has been shown, with the apoptotic effect of dopamine being higher than that of dobutamine (37). The implication of this finding for the benefit-risk analysis can only be assessed in the light of long-term follow-up.

The safety of dobutamine in the term infant, either with hypotension or low SVC flow, has not been tested in RCTs.

The Summary of Product Characteristics of commercially available dobutamine includes a listing of adverse events reported during exposure to dobutamine in adults. The following data relates to adults and may be relevant to neonates.

Evaluation of undesirable effects is based on the following frequency scale:

- Very common: ≥ 1/10
- Common: ≥ 1/100 to 1/10
- Uncommon: ≥ 1/1,000 to 1/100
- Rare: ≥ 1/10,000 to 1/1,000
- Very rare: < 1/10,000

- Not known: cannot be estimated from the data available

Blood and lymphatic system disorders

Common: Eosinophilia, inhibition of thrombocyte aggregation (only when continuing infusion over a number of days).

Metabolism and nutrition disorders

Very rare: Hypokalaemia.

Nervous system disorders

Common: Headache.

Cardiac disorders / vascular disorders

Very common:

Increase of the heart rate by ≥ 30 beats/min.

Common: Blood pressure increase of ≥ 50 mmHg. Patients suffering from arterial hypertension are more likely to have a higher blood pressure increase.

Blood pressure decrease, ventricular dysrhythmia, dose-dependent ventricular extrasystole.

Increased ventricular frequency in patients with atrial fibrillation.

These patients should be digitalised prior to dobutamine infusion.

Vasoconstriction in particular in patients who have previously been treated with beta blockers.

Anginal pain, palpitations.

Uncommon: Ventricular tachycardia, ventricular fibrillation.

Very rare: Bradycardia, myocardial ischaemia, myocardial infarction, cardiac arrest.

Not known: Decrease in pulmonary capillary pressure.

Children: Pronounced increase of heart rate and/or blood pressure as well as a lower decrease of the pulmonary capillary pressure than adults. Increase of pulmonary capillary pressure in children under 1.

Respiratory system, thoracic and mediastinal disorders

Common: Bronchospasm, shortness of breath.

Gastrointestinal disorders

Common: Nausea.

Skin and subcutaneous tissue disorders

Common: Exanthema.

Very rare: Petechial bleeding.

Musculoskeletal and connective tissue disorders

Common: Chest pain.

Renal and urinary disorders

Common: Increased urgency at high dosages of infusion.

General disorders and administration site conditions

Common: Fever, phlebitis at the injection site.

In case of accidental paravenous infiltration, local inflammation may develop.

Very rare: Cutaneous necrosis

Further undesirable effects

Restlessness, nausea, headache, paraesthesia, tremor, urinary urgency, feeling of heat and anxiety, myoclonic spasm.

2.4.2 Known Potential Benefits

Dobutamine is a sympathetic amine that increases myocardial contractility through direct stimulation of myocardial adrenergic receptors. This pharmacodynamic effect has been exploited by neonatologists for several decades and was associated with long-term benefit in a study with an active comparator (dopamine) (30).

Dobutamine may have other effects that would be beneficial. These include systemic and pulmonary vasodilatation. Vasodilatation and reduced vascular resistance would lead to improved blood flow organ distribution. Increased stroke volume can be accomplished with no change or even decreased blood pressure due to the drug´s potential vasodilatatory effects (38).

Alternative therapies are in use. Dopamine is the most commonly used therapy and is under investigation in a parallel drug development plan.

Contrary to dopamine, dobutamine does not affect plasma catecholamine levels. In contrast to dopamine, no suppression of the hypothalamus-pituitary axis has been found after dobutamine treatment in neonates (27).

Dobutamine has been found to be more effective than dopamine in increasing systemic blood flow but not blood pressure in the immature preterm neonate during the first hours of extra-uterine life (28).

The use of a combination of biomarkers that have been proven to be as good predictors for adverse outcome will better define the population at risk of end-organ damage due to abnormal blood flow distribution that eventually would benefit from dobutamine treatment.

2.4.3 Assessment of Potential Risks and Benefits

The significant therapeutic benefits and fulfilment of therapeutic needs outweigh the risks involved. The existent literature supports the further development of dobutamine as a treatment for haemodynamic insufficiency in the 72 hours after birth in infants born at 32(+6) weeks gestation or below, as long as that evaluation is embedded in studies that carefully document information relating to safety, efficacy and dosing.

There is a favourable benefit-risk balance to support the evaluation of dobutamine in carefully monitored clinical studies. There is no risk related to proposed procedures included in this protocol.

Efficacy:

- Dobutamine has pharmacodynamic effects in several species from animal experimentation and in newborn humans that support the use of dobutamine to treat haemodynamic insufficiency during adaptation to preterm birth.
- There is preliminary evidence in human neonates with haemodynamic insufficiency that dobutamine has beneficial short-term effects.

- The indications for dobutamine remain unclear
- The optimal way to monitor the effects of dobutamine remains unclear
- The optimal dose and triggers for dose escalation remain unclear

Safety:

- A range of adverse events are associated with dobutamine use in adults. These do not preclude the use of dobutamine in that population
- The data from animal experimentation and newborn humans does not add significantly to the safety concerns.

Investigators are reminded of the need to collect data relating to both efficacy and safety in the clinical study. This will underpin a comprehensive assessment of the benefit/risk ratio in neonates at the conclusion of this programme.

A weight of evidence (WoE) approach, following the ICH S11 guideline, supports that this paediatric plan does not need further preclinical studies. Although the paediatric age group refers to neonates, therefore making further studies likely, the expected duration of the clinical intervention (treatment) is very low (less than 72 hours in more than 90% of treated infants)(35); and the indication is an acute condition (not a chronic use). These facts make highly improbable that such a short exposure to a pharmaceutical can eventually harm developing organ systems. The existing clinical data relevant for the intended study population come from the same age range, which supports additional safety profile. Risk mitigation strategies are also defined in the study protocol (see sections 6.1.3 Individual and Global Safety Parameters; 6.2.1 Rescue Treatment).

3 OBJECTIVES AND ENDPOINTS

OBJECTIVES	ENDPOINTS
Primary	Primary
• To determine the minimum effective dose of dobutamine required to treat low SVC flow (<51 ml/k/min) in very preterm infants **(short- term pharmacodynamic (PD) objective).**	• Short-term PD endpoint: Minimum dobutamine dose to reach and maintain an SVC flow above 55 ml/k/min on an echocardiogram performed at 1 and 3 hours after effective infusion of the allocated dose [A].

Secondary	
• To assess the proportion of infants who maintain an acceptable haemodynamic status with the dobutamine infusion alone in the first 72h from birth (efficacy).<br>• To evaluate the safety of dobutamine for the whole study population as well as for the seven treatment groups separately.	• Proportion of neonates achieving and maintaining a clinically acceptable haemodynamic status [B] with the dobutamine infusion alone in the first 72 hours from birth.<br>• Absolute and relative frequencies of adverse events (AEs) and severe adverse events (SAEs), to be recorded and compared between groups.

• To determine the individual variables that explain the interindividual pharmacokinetic (PK)/PD variability.	• To determine the correlation between PK and PD.<br><br>[A] The effective start of the infusion (t0) will be calculated as the time at which the infusion pump is switched on plus the empirical value for the interval arising from the dead space. We summarize t0 as "the time at which dobutamine is expected to reach the circulation"<br><br>[B] Acceptable haemodynamic status is defined, for the purposes of this study, as the achievement and maintenance of dose success during the first 72 h from birth. The loss of such acceptable haemodynamic status occurs whenever there is a change in therapeutic strategy that involves cardiovascular treatment other than dobutamine alone due to exceeded safety parameters, treatment failure of the investigational infusion or the need for rescue treatment or death; any additional fluid bolus is considered as cardiovascular treatment.

4 STUDY DESIGN

4.1 OVERALL DESIGN. LOW-INTERVENTION CLINICAL TRIAL.

NeoCirc-002 is a single center, dose finding trial to define the minimum effective dose of dobutamine to treat early hemodynamic insufficiency in the preterm infant (less than 33 completed weeks' gestation), defined as presence of low SVC flow (<51 ml/k/min) within the first 72 hours from birth.

The trial is a low-intervention clinical trial since dobutamine is the standard treatment for circulatory failure in preterm infants during transitional circulation, the dosages studied are within the standard therapeutic range, and the diagnostic supplementary procedures are in routine clinical practice.

4.2 SCIENTIFIC RATIONALE FOR STUDY DESIGN AND DOSE

As other catecholamines, dobutamine is always administered in continuous i.v. infusion, preferably through a central line.

Recent data from clinical and pre-clinical investigations, and clinical experience, indicate that dobutamine can be safely up titrated from a starting dose of 5 µg/kg/min in steps of 5 µg/kg/min in rather flexible timing within limits of up to 20 µg/kg/min; and that there is until now no evidence that dobutamine could introduce harm in newborn babies using this treatment scheme (11, 28, 41).

Although the information is scarce, there is a wide range in threshold doses of dobutamine needed to increase heart rate, blood pressure or cardiac output in children and adolescents (31-33), so that individual titration is extremely important. The minimum dobutamine dose at which a pharmacodynamic effect has been shown in neonates is 10 µg/kg/min (15-17, 19, 23, 25, 26,

28). In two RCT conducted in preterm infants on the effect of dobutamine on SVC flow (11, 28), the lowest dobutamine dose that was evaluated was 10 µg /kg/min.

Results from PK sub-study NeoCirc-001A revealed high inter-individual PK variability regarding dobutamine elimination half-life and consequently, time to reach steady-state that ranged from 15 min to 180 min (35). Due to this important inter-individual variability, there is still a need to confirm the lower effective dose in very preterm neonates; therefore, the proposed study will cover dobutamine dose-range from 5 to 20 µg /kg/min.

4.4 END OF STUDY DEFINITION

An infant is considered to have completed the study if he or she has completed all phases of the study including the last visit or the last scheduled procedure shown in the Schedule of Activities (SoA), Section 1.3.

The end of the study is defined as completion of the last visit or procedure shown in the SoA in the trial.

5 STUDY POPULATION

5.1 INCLUSION CRITERIA

Infants are eligible to be included in the study only if they meet **all** the following criteria:

- Born up to 32(+6) weeks gestation
- Presence of haemodynamic insufficiency, defined as SVC flow <51 ml/kg/min.
- Provision of signed and dated informed consent form by father/mother or legally designated representative, which can be given antenatally as described in section 5.4.

Three notes of caution are necessary with respect to inclusion of infants into this trial:

- The treating physician needs to be at "clinical" equipoise on providing the proposed trial of dobutamine-treatment to the infant, which can be justified as explained in section 2.1 above.
- To avoid over treatment, the treating physician needs to fully understand the determinant of the pathophysiology of circulatory impairment and use clinical judgment to decide treatment. For example, during severe congenital anaemia a baby meeting the inclusion criteria will not be included and will instead get the required blood volume replacement first; and only afterwards could be considered for inclusion if still eligible.
- Deferred informed consent and prior informed assent (opt-out with enrollment as default) are plausible in this condition because it is in accord with Article 35 of the Regulation of the European Union on Clinical Trials (Regulations (EU) no536/2014), that states: Informed consent to participate in a clinical trial may be obtained, and information on the clinical trial may be given, after the decision to include the subject in the trial, provided that this decision is taken at the time of the first intervention on the subject, in accordance with the protocol for that clinical trial and that all the following conditions are fulfilled:
    - Urgency of the situation, caused by a sudden life-threatening or other serious medical condition
    - Scientific grounds to expect that participation of the subject in the trial will have the potential to produce a direct clinically relevant benefit for the subject resulting in a measurable health-related improvement or in the diagnosis of its condition

- It is not possible within the therapeutic window to supply all prior information to and obtain prior informed consent from his/her legally designated representative
- The investigator certifies that he or she is not aware of any objections to participate in the clinical trial previously expressed by the legally designated representative
- The clinical trial relates directly to the subject's medical condition because of which it is not possible within the therapeutic window to obtain prior informed consent from the legally designated representative and to supply prior information, and the clinical trial is such a nature that it may be conducted exclusively in emergency situations
- The clinical trial poses a minimal risk to, and imposes a minimal burden on, the subject in comparison with the standard treatment of the condition.

Informed consent shall be sought to continue the participation of the subject in the clinical trial, and information on the clinical trial shall be given to the parent or the legally designated representative.

5.2 EXCLUSION CRITERIA

Patients will be excluded from the study if they meet any of the following criteria:

- Neonates considered non-viable, with a clinical decision not to provide life support
- Infants with severe congenital hydrops fetalis needing chest or peritoneal drainage before recruitment
- Infants already on dobutamine treatment
- Infants with congenital malformations likely to affect cardiovascular adaptation (including: congenital diaphragmatic hernia, gastroschisis or congenital heart defects).
- Infants carrying chromosomal anomalies
- Lack of parental signed informed consent

5.3 SCREEN FAILURES

Screen failures are defined as participants whose father/mother or legally designated representative consent to participate in the clinical trial AND undergo screening assessments to see if they can enter the study, BUT are not subsequently randomly assigned to the study intervention or entered in the study. The data from the Screening Visit should be recorded into the eCRF for the screen failures.

5.4 STRATEGIES FOR RECRUITMENT AND RETENTION

By definition, this is a trial conducted on a vulnerable population, both from the perspective of the subject but also from the point of view of the parents. In many instances, preterm birth is produced suddenly, without an anticipated cause. Even in the case of antecedents supporting the risk, parents are never prepared for a preterm delivery. In particular, the most immature preterm infants will need intensive care treatments to help their breathing, and many of them will need interventions to improve their circulation soon after birth.

This is a stressful time for parents and families. Coping with all the information provided at this early stage is difficult for parents. For this reason, a strategy for recruitment is in place to avoid

overloading parents with information during the critical period after birth when they are most vulnerable:

- Whenever possible parents will give written informed consent antenatally after explanation of the aims, methods, benefits and potential hazards of the trial. The implications of the trial to the neonates enrolled should be clearly explained by the local research team as part of the informed consent process.
- Parents that have not yet given written informed consent for the trial will be approached as soon as possible after the birth of a baby between a gestational age of 24 weeks (+0 days) and 32 weeks (+6 days) and postnatal age <72 hours in risk of haemodynamic insufficiency; and provided with the relevant parental information.

With this strategy many parents would have already given signed informed consent antenatally.

Only those babies with existing signed informed consent can be enrolled into the trial (randomized and receive the trial intervention).

Given that the condition can be considered as an emergency in some situations, and treatment should be indicated if circulatory failure is present regardless of the clinical trial, deferred informed consent and prior informed assent (opt-out with enrollment as default) would be applicable, in accord with Article 35 of the Regulation of the European Union on Clinical Trials (Regulations (EU) no536/2014) (see section 5.1). However, it is worth noting that deferred consent and opt-out are only allowed in a handful of very specific situations that will need to be fully described at a site level in agreement with the PI and local hospital policies.

6 STUDY INTERVENTION

6.1 STUDY INTERVENTION ADMINISTRATION

6.1.1 Study Intervention Description

Once the patient has fulfilled the inclusion criteria, he or she will be allocated to a dosage regimen according by the study statistician. The statistician will be informed about the therapeutic response observed for re-estimation of the posterior probability of success after each cohort of 3 patients. The choice of allocation will be decided according to dose with the posterior probability closer to the target probability (80%).

The dobutamine solution is commercially available dobutamine (Dobutamine Generis, concentration 12.5 mg/mL). It will be impossible to blind carers or assessors to the dose used.

6.1.2 Dosing and Administration

The study medication for infusion will be prepared and administered with strict standardisation of the administration system to minimise systematic errors due to differences in infusion set-ups. This will ensure the precise time at which dobutamine enters the body can be recorded (t0).

The infant's treatment will be guided by the responsible physician who will use the study medication under the premise of keeping a minimum time of 60 minutes from the effective infusion of dobutamine (t0) for PD assessment.

Once the primary PD variable has been assessed further management will be at the discretion of the attending physician. Therefore, if there is no PD response according to protocol with the allocated starting dose, or even if there were, the clinical situation of the patient so recommends it, the responsible physician decide on further treatment (Rescue treatment in section 6.2.1). However, clinicians are encouraged to consider the up-titration to maximum dobutamine dose first, to try to maintain the infant in the study drug as long as possible, if it is the best option for the infant.

Any changes to the treatment strategy will be documented by the responsible physician.

6.1.3 Individual and Global Safety Parameters

In addition to rescue treatment in the absence of a clinical response to the study intervention, as described in section 6.4.1, the following safety parameters have been established to safeguard infants participating in the clinical trial.

A change of dose is allowed during the waiting time for clinical response, if any of the following **Individual Safety Parameters** are exceeded:

- Heart rate rises above 200 bpm.
- Mean blood pressure rises above pre-established values (Table 4)

Table 4. Accepted upper limits of blood pressure according to GA and day of life

	Day of life 1			Day of life 2			Day of life 3		
**GA**	**Systolic**	**Diastolic**	**Mean**	**Systolic**	**Diastolic**	**mean**	**Systolic**	**Diastolic**	**Mean**
**24**	46	33	37						
**26**	50	35	40						
**28**	55	37	43						
**<28**				50	36	41	52	37	42
**30**	58	39	45						
**32**	60	41	47						
**33**	63	43	49						
**28-32**				61	44	50	65	45	52

In case of having a dose evaluation out of schedule (outside the 60 minutes intervals) due to a safety alert that triggers a dose modification, the infusion start time of the newly modified dose will be considered the 0 min time for a new 60 minutes dose evaluation.

The following **Global Safety Parameters** will also permit a change of dose during the waiting time for clinical response and may trigger a toxicity alert and halt of the trial in one or more clinical sites:

- Expected percentage of patient presenting safety issues:
  - Blood pressure above individual safety thresholds: < 20%
  - Heart rate above safety threshold: <15%

6.2 CONCOMITANT THERAPY

There is no specified 'per-protocol' concomitant medication or treatment. Any drugs other than those listed in section 6.3.2 are allowed, and should be administered, as necessary for the treatment of the patient. Only concomitant cardiovascular treatments, such as other catecholamines, hydrocortisone or others, should be documented on the appropriate eCRF. Fluid boluses are considered also cardiovascular treatment and should be recorded on the eCRF.

All allowed medications that have been started before screening may be continued during the study. Medications for treatment of emerging illnesses that arise after screening will be allowed. The medication and the condition must be documented on the adverse event page of the eCRF (when applicable, please refer to the assessment of safety section of the protocol). For any concomitant therapy given as a treatment for a new condition or a worsening of an existing condition, the condition must be documented on the adverse event page of the eCRF (when applicable, please refer to the assessment of safety section of the protocol).

6.2.1 Rescue Treatment

A change in treatment strategy using rescue treatments is allowed if no clinical response to the study intervention is considered by the attending physician, when the

- MABP< GA-5 mmHg

and one or more of the following:

- SVC flow < 51 ml/kg/min (after 1 hour)
- Rising serum lactate
- Base excess being more negative
- CRT≥4sec

Rescue treatments include:

- Additional fluid bolus
- vasopressor-inotrope therapy, apart from commercial dobutamine
- corticosteroids
- other treatment administered to treat haemodynamic insufficiency

When rescue treatment is needed physicians will be encouraged to consider up-titration option first, to try to maintain the infant in the study drug only treatment corridor as long as possible, if it is the best option for the infant. Rescue treatment will not include commercial dobutamine.

6.2.2 Precautionary Medications, Treatments and Procedures

- Entacapone: This drug is a selective inhibitor of the catechol-o-metil transferase. Actions and side effects may be increased by dobutamine.
- Beta-blockers: The inotropic effect of dobutamine stems from stimulation of cardiac beta1 receptors, this effect is reversed by concomitant administration of beta-blockers. Dobutamine has been shown to counteract the effect of beta-blocking drugs. In therapeutic doses, dobutamine has mild alpha1- and beta2-agonist properties. Concurrent administration of a non-selective beta-blocker such as propranolol can result in elevated blood pressure, due to alpha-mediated vasoconstriction, and reflex bradycardia. Beta-blockers that also have alpha-blocking effects, such as carvedilol, may cause hypotension during concomitant use of dobutamine due to vasodilation caused by beta2 predominance (see section 4.4 Special warnings and precautions for use).
- Alternative fluid bolus (only allowed as rescue medication)
- Vasopressor-inotrope therapy (only allowed as rescue medication)

6.2.3 Prophylactic Medications, Treatments and Procedures

There will not be, per protocol, any prophylactic medication, treatment, or procedure. Overall care of the infant will follow the policies currently in use in the unit.

7 STUDY INTERVENTION DISCONTINUATION AND PARTICIPANT DISCONTINUATION/WITHDRAWAL

7.1 DISCONTINUATION OF STUDY INTERVENTION

Discontinuation from commercial dobutamine does not mean discontinuation from the study, and remaining study procedures should be completed as indicated by the study protocol. If a clinically significant finding is identified after enrolment, the investigator or qualified designee will determine if any change in participant management is needed. Any new clinically relevant finding will be reported as an adverse event (AE).

The criteria for enrolment must be followed explicitly. If a patient who does not meet enrolment criteria is inadvertently enrolled, that patient is to be discontinued from the study drug but observation should be continued according to the study protocol in order to provide the follow-up data needed for the analysis of the entire intention-to-treat population, to which the patient belongs formally.

In addition, patients will be discontinued from study treatment in the following circumstances:

a) The investigator decides that the patient should be withdrawn from study treatment. If this decision is made because of a serious AE (SAE), the study drug is to be discontinued, and appropriate measures are to be taken. The Sponsor or its designee is to be notified immediately.
b) The infant's parent(s)/legal representative, for any reason, requires treatment with another therapeutic agent that has been demonstrated to be effective for treatment of the study indication. In this case, discontinuation from study treatment should occur immediately upon introduction of the new agent.

Investigator decides to withdraw the patient: Patients who discontinue the study drug early will have the Last Treatment Assessment (LTA) Visit procedures performed as shown in the SoA (section 1.3). Discontinuation of study treatment does not necessarily mean that observation in line with the protocol needs to end at this point in time and, unless indicated from the treating physician's opinion, should proceed in line with the study plan.

Legal representative decision: Infant´s parents/ legal representative will be asked whether they will consent the infant to remain in the trial for the remaining study- and follow-up assessments and inclusion of his/her already collected data in a database, a registry, or a publication. All efforts will be made to complete and report the observations as thoroughly as possible up to the date of withdrawal. All information should be reported on the applicable CRFs.

Withdrawal of partial consent means that the participant's legal representatives do not wish the participant to take investigational medical products any longer but are still willing to collaborate in providing further data by continuing with the study (i.e., participating in subsequent study visits or procedures and/or provide overall survival information).

7.2 PARTICIPANT DISCONTINUATION/WITHDRAWAL FROM THE STUDY

A participant may be withdrawn from the study at any time at the request of his/her parent(s), or may be withdrawn at any time at the discretion of the investigator or sponsor for safety or administrative reasons.

If a participant withdraws from the study, he/she may request destruction of any samples taken and not tested, and the investigator must document this in the site study records.

The Last Treatment Assessment (LTA) Visit data should be collected at the time of study discontinuation and the reason for participant discontinuation or withdrawal from the study will be recorded on the early discontinuation form of the eCRF.

Subjects who sign the informed consent form and are randomized but do not receive the study intervention will be excluded from the Intention-To-Treat (ITT) population and may therefore be replaced. There will be no replacement of subjects who withdraw after they have been enrolled in the study and received at least one dose of study drug.

7.3 LOST TO FOLLOW-UP

Given the shortness of the study observation it is highly improbable to have such events.

We only envisage a potential source of lost to follow-up of enrolled infants, consisting on early discharge/transfer to lower care unit (before 34 weeks) which is rather infrequent. In these cases, the research team are committed to contact the families/transfer hospital for a required study visit. Should the participant continue to be unreachable, he/she will be considered to have withdrawn from the study with a primary reason of lost to follow-up.

8 STUDY ASSESSMENTS AND PROCEDURES

8.1 EFFICACY ASSESSMENTS

The following sections describe all procedures and evaluations to be done as part of the study.

The specific timing of procedures/evaluations at each study visit is described in Section 1.2, Schema and Table.

8.1.1 Study Visits Procedures

8.1.1.1 Screening Visit

During the Screening Visit, potentially eligible infants are screened for inclusion/exclusion within 0-72h of birth. Potential candidates for enrolment are infants with haemodynamic insufficiency in the first 72 hours after birth, born at less than 33 weeks gestation (echocardiography will be performed in the first 24h and every 10-12h during the first 72h after birth). Eligibility parameters (SVC flow <51 ml/kg/min) as well as other clinical and biochemical parameters are assessed if clinically indicated but not all of them will be recorded in the eCRF.

Strategies for recruitment outlined in section 5.4 and the informed consent process described in section 10.1.1 are followed during the screening and enrolment of patients into the study, using specially designed Informed Consent Documents (ICD).

8.1.1.2 Enrolment: Baseline Visit

If the inclusion criteria are met, and there are no reasons for exclusion, the infant is then enrolled into the trial and allocated to a dosage regimen according by the study statistician. At this point the Screening Visit finishes and the Baseline Visit parameters outlined in section 8.1.2.3 below are assessed.

The study intervention should be initiated straight away after the Baseline Visit always within the first 72 hours from birth.

8.1.1.3 Treatment: Start of Dobutamine Infusion Visit

For all dosage regimen, the study intervention is initiated with continuous infusion of the initial dose of dobutamine during the first treatment visit.

8.1.1.4 Treatment: PD/PK Assessment Visit

The patient PD response to the dobutamine treatment should be evaluated at 60 minutes from the start of effective dose infusion, to establish if the treatment success dose has been achieved. Blood sample (1 millilitre) will be taken.

Effective dose infusion is define as the time (t_0) when the study medication enters blood stream, which is calculated according to the following equation:

$t_0 = t_{switch\ on} + t_d$

td (min)= (volume (ml)/ velocity (ml/h)) x 60 (min/h),

where *volume* refers to the catheter lumen plus the stopcock dead space and *velocity* refers to the summation of the infusion rate of all the infusions coming through the line. Dobutamine infusion should be at the stopcock closest to the catheter entry. Before dobutamine is connected to the stopcock, the whole system (including bionector) will be fed with the dobutamine solution and the pump will be switched on for a few minutes to ensure that no pulling out effect is added at start. The pump will be switched off and the system will be connected to the stopcock. Finally the pump will be switched on and the time will be registered ($t_{switch\ on}$)

The following criteria will be checked at every patient at the dose evaluation:

- Main response biomarker: SVC flow > 55 ml/kg/min

And other parameters:

- MABP increase by 20% (roughly 4-6 mmHg)
- Decreasing serum lactate levels
- Base excess levels being less negative
- CRT < 4 sec

Throughout the treatment, a series of clinical and biochemical parameters described in sections 8.1.2 below are evaluated and recorded in the eCRF according to the timings given in the section 1.3.

8.1.1.5 Treatment: PD/ PK re-assessment Visit

The patient PD response to the dobutamine treatment should be re-evaluated at 180 minutes from the start of infusion of the effective dobutamine dose (SVC flow above 55 ml /K/min). A second one millilitre of blood sample will be obtained.

8.1.1.6 Efficacy Visit

To test whether dobutamine alone achieved and maintained an acceptable haemodynamic status during the first 72 h from birth (secondary objective).

This study short-term efficacy outcome means that at 72h the infant is alive, and the clinical condition is:

- SVC flow > 55 ml/kg/min;
- MABP > GA;
- Decreasing serum lactate levels;
- CRT < 4s;
- Urine output > 1 ml/kg/h;
- Dobutamine alone was used (regardless the dose).

Investigators will be asked to report if, according to this definition, short-term success has been achieved or not. If not, investigators will also be asked to check if persistent circulatory failure or death is due to something different from pure transitional circulation adaptation, e.g., sepsis, haemodynamically significant PDA, air trapping (lung over distension), pneumothorax, pleural or pericardiac effusion.

8.1.1.7 End of Treatment Assessment:

Weaning and stopping of the dobutamine infusion will be at the criteria of attending physician, proceeding as done in clinical practice for other catecholamine treatment and following local policies. Therefore, no set guidance is provided per protocol, although it is recommended to follow the same down-titration scheme as up-titration.

The End of Treatment Assessment Visit occurs at treatment completion, when the dobutamine infusion has been stopped.

8.1.1.8 Mid and Long-term assessment.

Visit at 36±2 weeks PMA to record main neonatal clinical outcomes, included bronchopulmonary dysplasia, necrotizing enterocolitis, retinopathy of prematurity and neurological complications (cerebral haemorrhage grade II or III, periventricular haemorrhagic infarction, white matter injury, post-haemorrhagic ventricular dilatation, cerebellar haemorrhage or cerebral atrophy).

Long-term follow up of the study population will take place at 24 (±4) months of age corrected for prematurity. However, it is not feasible to precise which will be the first patient assessed because will depend on prematurity. We provide 28-months period to complete the long-term follow up of the whole study population. Within this timeframe, there is margin for a complete follow up even for the latest patients enrolled irrespective of their immaturity (how low the gestational age was at birth). The follow up will include growth assessment (weight, height and head circumference), general health, structured neurological examination and neurodevelopmental testing.

8.1.2 Basic Evaluations

The procedures and evaluations described in this section will be undertaken according to the timing described in the SoA table (Section 1.3).

8.1.2.1 Medical History

The following data will be captured from the participant's clinical records:

- Demographic data: date of birth and gender
- Maternal data: Antenatal corticosteroid use (complete course); Maternal hypertension and treatment; Chorioamnionitis: fever (>38ºC), foul smelling amniotic fluid, tenderness of the uterus, maternal/foetal tachycardia, maternal leukocytosis, histo-pathological diagnosis; Prolonged rupture of membranes (>24 hours to delivery); Type of delivery; Any other relevant condition.
- Perinatal data: Gestational age; Multiple (order); Need for advanced resuscitation at birth (intubation± assisted ventilation); Placental transfusion procedure (delayed cord clamping, milking of the cord); Birth weight; 5 min Apgar score; SNAPPE-II score (42); Surfactant.
- Neonatal outcomes: overall mortality; cUS diagnoses; Necrotising enterocolitis or bowel perforation; bronchopulmonary dysplasia (oxygen dependency at 36 week' gestation); early infection (positive cultures or CRP <72h of birth); nosocomial infection (use of antibiotics for at least 5 days regardless of negative or positive blood culture); patent ductus arteriosus (PDA) and treatment; retinopathy of prematurity (ROP) and treatment, discharge date, discharge type (home, transfer to other hospital).

8.1.2.2 Medication

The maximum dobutamine dose infusion in the first 72h from birth will be recorded in the eCRF.

All co-existent diseases or conditions will be treated in accordance with prevailing medical practice. All allowed medications that have been started before screening may be continued during the study (see also section 6.3).

The need for additional cardiovascular support to that specified for the clinical trial will also be recorded.

8.1.2.3 Clinical and Biochemical Parameters

The clinical and biochemical parameters listed next will be recorded and entered into the eCRF at the study time points indicated in section 1.3 (SoA):

• Physical and vital signs: heart rate; blood pressure (systolic, mean and diastolic); pulse oximetry (SpO2); capillary refill time (CRT); and type of mechanical ventilation (CPAP, MV, HFOV, HFNC; none).

• Biochemical parameters (from clinically indicated blood samples): pH; pCO2; lactate; base excess; haemoglobin; creatinine; C-reactive protein; glucose. The volume of blood required for these samples is 0.3 ml to 0.5 ml, approximately.

• Urine output every 4 hours.

• SVC flow and RVO as clinical parameters: echocardiography (Echo-D) will be performed as soon as possible after birth, during screening phase, and during treatment phase for PD assessment of the study medication. At each Echo-D scan, SVC flow and RVO will be measured and recorded in the eCRF as indicated in SoA table (section 1.3). SVC flow will be measured using the traditional technique of Kluckow & Evans (29) which assesses SVC diameter from a parasternal long-axis view and flow velocity from low subcostal view by averaging 5-10 cardiac cycles (43).

• Routine evaluation for PDA is not mandated per protocol at each Echo-D; however, PDA assessment will be done when clinically indicated.

8.1.2.4 Cranial Ultrasound

Cranial ultrasound studies (cUS) will assess neurological complications for the medium-term efficacy assessment. Severe neurological complications are defined based on worst cranial ultrasound (cUS) scan appearances detected on any one of a series of cUS scans that are routinely performed in these infants up to 36±2 weeks PMA. Severity of cUS imaging is to be graded using a modified score published by the SafeBoosC trial (1-4). Severe brain injuries will be cerebral haemorrhage grade II or III, periventricular haemorrhagic infarction, white matter injury, post-haemorrhagic ventricular dilatation, cerebellar haemorrhage, or cerebral atrophy.

It is strongly recommended to perform a first cUS scan as soon as possible at screening, preferably within the first 6 hours from birth and before treatment is started. Subsequent scans will be done when clinically indicated.

Only two cUS proformas should be filled in: baseline cUS performed during the screening phase (if available); and worst cUS findings detected on any of cUS scans up to 36±2 weeks PMA.

8.1.2.5 Pharmacogenetic

To analyse the pharmacogenetics of GNAS1 and β1-adrenoceptor polymorphism and different metabolizer enzymes of dobutamine we will be gained buccal swabs.

The scraping samples of the oral mucosa will be obtained at enrolled in the study by the nursery staff. A cotton swab will be applied gently to both cheeks up and down with a double-tipped, sterile swab. The buccal swab will be removed and placed back in the sterile tube.

8.1.2.6 Standard of Care Study Procedures

There will be no per-protocol concomitant medication or treatment. Accordingly, other important co-interventions will follow specific centre protocols and will be recorded in the case record form (eCRF).

All trial related procedures will be undertaken in line with local procedures to minimise pain and optimise welfare. All trial-related procedures will be conducted by professionals experienced in neonatal care who have received trial-specific training. Non-pharmacological (behavioural) and pharmacological analgesic measures (sucrose, paracetamol, and opiates) will be preferably used in accordance with local guidelines and the condition of the baby at the time of the procedure. Blood sample volumes will be minimised.

8.2 SAFETY AND OTHER ASSESSMENTS

The safety of the study intervention will be evaluated, as a secondary endpoint, by recording and comparing the absolute and relative frequencies of Adverse Events (AEs) and Serious Adverse Events (SAEs) for the whole study population and for the five treatment groups.

Pharmacovigilance definitions and processes for this purpose are defined in section 8.3, while the intended statistical analysis of safety as a secondary endpoint is described in section 9.1.2.

8.3 ADVERSE EVENTS AND SERIOUS ADVERSE EVENTS

8.3.1 Definition of Adverse Events (AE)

Adverse Event (AE):

Any untoward medical occurrence in a patient or clinical investigation patients administered a medicinal product, which does not necessarily have a causal relationship with this treatment (the study medication).

An AE can therefore be any unfavourable and unintended sign (including an abnormal laboratory finding), symptom or disease temporally associated with the use of the study medication/procedure, whether considered related to the study medication.

This includes any newly occurring event, or a previous condition that has increased in severity or frequency since the administration of study drug.

Adverse Reaction (AR):

All untoward and unintended responses to a medicinal product related to any dose administered.

Unlike Adverse Events, a causal relationship between a study medication and an AE is considered at least a reasonable possibility, i.e., the relationship cannot be ruled out.

8.3.2 Definition of Serious Adverse Events (SAE)

Serious Adverse Event (SAE) or Serious Adverse Reaction (SAR):

A serious adverse event is any untoward medical occurrence that at any dose:

- Results in death,
- Is life-threatening. (NOTE: The term "life-threatening" in the definition of "serious" refers to an event in which the participant was at risk of death at the time of the event; it does not refer to an event which hypothetically might have caused death if it were more severe).
- Requires inpatient hospitalization or prolongation of existing hospitalization,
- Results in persistent or significant disability/incapacity, or
- Is a congenital anomaly/birth defect.
- Other important medical events. NOTE: Other events that may not result in death are not life threatening, or do not require hospitalization, may be considered a serious adverse event when, based upon appropriate medical judgment, the event may jeopardize the patient and may require medical or surgical intervention to prevent one of the outcomes listed above.

To ensure no confusion or misunderstanding of the difference between the terms "serious" and "severe", which are not synonymous, the following note of clarification is provided:

The term "severe" is often used to describe the intensity (severity) of a specific event (as in mild, moderate, or severe myocardial infarction); the event itself, however, may be of relatively minor medical significance (such as severe headache). This is not the same as "serious," which is based on patient/event outcome or action criteria usually associated with events that pose a threat to a participant's life or functioning as defined in the bullet points above. Seriousness (not severity) serves as a guide for defining regulatory reporting obligations.

8.3.3 Classification of an Adverse Event

8.3.3.1 Severity of Event

The following guidelines will be used to describe severity of the adverse events (AEs):

- **Mild** – Events require minimal or no treatment.
- **Moderate** – Events result in a low level of inconvenience or concern with the therapeutic measures. Moderate events may cause some interference with functioning. Some assistance may be needed, no or minimal medical intervention.
- **Severe** –May require systemic drug therapy or other treatment. Severe events are usually potentially life-threatening or incapacitating. Of note, the term “severe” does not necessarily equate to “serious”.

8.3.3.2 Relationship to Study INTERVENTION

All adverse events (AEs) must have their relationship to study intervention assessed by the clinician who examines and evaluates the participant based on temporal relationship and his/her clinical judgment. The degree of certainty about causality will be graded using the categories below. In a clinical trial, the study product must always be suspect.

- **Related** – The AE is known to occur with the study intervention, there is a reasonable possibility that the study intervention caused the AE, or there is a temporal relationship between the study intervention and event. Reasonable possibility means that there is evidence to suggest a causal relationship between the study intervention and the AE.
- **Not Related** – There is not a reasonable possibility that the administration of the study intervention caused the event, there is no temporal relationship between the study intervention and event onset, or an alternate etiology has been established.

8.3.3.3 Expectedness

Investigators at sites will be responsible for determining whether an adverse event (AE) is expected or unexpected. An AE will be considered unexpected if the nature, severity, or frequency of the event is not consistent with the risk information previously described for the study intervention.

Unexpected adverse reaction:

An adverse reaction, the nature or severity of which is not consistent with the applicable product information.

Suspected Unexpected Serious Adverse Reaction (SUSAR):

Serious adverse drug reaction (SAR) that is unexpected or for which the development is uncommon (unexpected issue) observed during a clinical trial and for which there is a relationship with the study drug.

These adverse drug reactions, characterized to be serious and unexpected, are mandatory to be communicated immediately in accordance with national legislation and international guidelines.

8.3.4 Time Period and Frequency for Event Assessment and Follow-Up

The investigator will monitor and systematically collect all the AEs from the time of **allocation** until **30 days after the patient has been stopped the administration of the drug**.

All AEs spontaneously reported by the patient parents and/or in response to an open question from study personnel or revealed by observation, physical examination, or other diagnostic procedures will be recorded in the history. Adverse events that were related with the study medications (AR and SAR) or not related but serious (SAE) will be collected on the appropriate page of the eCRF.

All adverse events will be monitored until they are resolved or stabilized. All adverse events that persist once the study is over will also be monitored until their final evolution is determined.

It will be left to the investigator´s clinical judgment whether an AE is of sufficient severity to require the participant´s removal from treatment. An infant's parents/legal representative may also voluntarily withdraw from treatment due to what he or she perceives as an intolerable AE. Whether either of these occurs, the participant must undergo an end of study assessment and be given appropriate care under medical supervision until symptoms cease or the condition becomes stable.

8.3.5 Adverse Event Reporting

All **Adverse Events (AEs)** that occur during the trial will be documented in the patient's medical history. AEs that were related with the study medications (AR and SAR) or not related but serious (SAE) will be collected on the appropriate page of the eCRF, the following information about the AEs will be recorded: description, start and end date, severity, seriousness, evaluation in relation to the investigation drug, actions taken and outcome.

Any pre-existent illness exacerbation that occurs after drug study initiation should be considered as an adverse event.

Any abnormal lab result that the investigator considers clinically important and that requires study drug dose adjustment, transitory of permanent study drug discontinuation or any other interventional measure or diagnostic evaluation for patient risk evaluation will be collected as an adverse event and should be investigated and monitored appropriately.

Serious Adverse Reaction (SAR) that occur during the trial must be **notified to the sponsor** within a maximum period of 24 hours from the time of knowing about the event.

Also, the investigator shall complete and sign the SAR report form and send it by fax or email to:

farmacovigilancia.ucicechulp@gmail.com

Responsible For Pharmacovigilance

Unidad Central de Investigación Clínica y Ensayos Clínicos (UCICEC)

Hospital Universitario La Paz

Paseo de la Castellana, 261

28046 Madrid

+34 912-07-14-66

The sponsor or whoever assumes the tasks delegated by the sponsor will review the received form and, if necessary, request additional information from the investigator. The investigator will provide information to the sponsor or whoever assumes the tasks delegated by the sponsor, whenever asked and, in all cases, when the initial assessment changes in severity or causality. To communicate the follow-up information, repeat the reporting procedure described previously.

The sponsor or whoever assumes the tasks delegated by the sponsor shall keep detailed records of all the SARs notified by the investigators.

9 STATISTICAL CONSIDERATIONS

9.1. STATISTICAL ANALYSES

9.1.1. Analysis of the Primary Efficacy Endpoint(s)

To calculate the dose-escalated sample size, the Bayesian Method of Continual Reassessment (46-49) was used, using the following a priori probability of success for each dose:

A. 5 mcg/kg/min → 45-50%
B. 7.5 mcg/kg/min → 70-75%
C. 10 mcg/kg/min → 80-85%
D. 12.5 mcg/kg/min → 90-95%
E. 15 mcg/kg/min → 95-100%

In addition, a target probability of 80%, maximum sample size of 30 patients and a confidence level of 90% was considered. The statistical software RStudio (version 4.1.1) and the web application of the Division of Translational Research and Applied Statistics, University of Virginia, were used.

Sample size: the estimated number of patients per dose is shown below:

	STEP 1	STEP 2	STEP 3	STEP 4	STEP 5
DOSAGE (mcg/kg/min)	5	7.5	10	12.5	15
EFFICACY (%)	45	70	80	90	95
PATIENTS (n)	5	9	12	4	0

After the simulation and at each dose-escalated, an implementation of the results obtained will be performed, recalculating the efficacy estimates per dose and the required number of patients. This escalation will stop when the expected efficacy is achieved.

9.1.2. Analysis of the Secondary Endpoint(s)

Efficacy:

The proportion of infants who maintain an acceptable haemodynamic status with the dobutamine infusion alone in the first 72h from birth.

Patients with a loss of acceptable haemodynamic status in the first 72 h from birth, but with confirmation that the underlying aetiology of circulatory failure is different from transitional circulation adaptation will be excluded. These include sepsis, haemodynamically significant PDA, air trapping (lung over distension), pneumothorax, pleural or pericardial effusion

Safety:

Safety analysis will be conducted for the whole study population as well as for the five treatment groups separately. AEs and SAEs will be reported with absolute and relative frequencies per treatment group along with the corresponding 95% CIs (two-sided). Differences between different groups (each of the dose regimens separately) will be compared using Chi-squared Test. For all comparisons 95% CIs (two-sided) will be provided.

Pharmacokinetic and pharmacogenetic analysis:

Pharmacokinetic analysis will be performed for the complete study population. Differences between different groups and patients will be compared using Chi-squared Test. For all comparisons 95% CIs (two-sided) will be required.

9.1.3. Baseline Descriptive Statistics

Demographic and baseline variables will be recorded for each group with mean, median and standard deviation for numerical variables and absolute and relative frequencies for categorical variables. Variables will be compared descriptively between each of the five dose regimen groups with mean differences and t-tests and rate differences and Chi-squared test respectively. 95%-confidence intervals (CIs) will be provided.

10. SUPPORTING DOCUMENTATION AND OPERATIONAL CONSIDERATIONS

10.1. REGULATORY, ETHICAL, AND STUDY OVERSIGHT CONSIDERATIONS

10.1.1. Informed Consent Process

10.1.1.1 Consent and Other Informational Documents Provided to participants

Consent forms describing in detail the study agent, study procedures, and risks are given to the parent/ patient’s legally acceptable representative and written documentation of informed consent is required prior to starting intervention/administering study product.

10.1.1.2 Consent Procedures and Documentation

The Investigator (according to applicable regulatory requirements), or a person designated by the Investigator (must be suitably qualified and experienced), and under the Investigator's responsibility, should fully inform the parent/ patient’s legally designated representative of all pertinent aspects of the clinical trial, including the written information given approval/favourable opinion by the Ethics Committee.

Prior to a patient's participation in the clinical trial, the written ICD should be signed, name filled in and personally dated by the parent/ patient's legally designated representative, and by the person who conducted the informed consent discussion. A copy of the signed and dated written ICD will be provided to the patient. The original signed form will be retained at the study site. As discussed on sections 5.1 and 5.3, deferred informed consent may apply to this trial and the ICD signed *a posteriori*, whenever the clinical situation is considered an emergency and the statements on *Article 35 of the Regulation of the European Union on Clinical Trials (Regulations (EU) no536/2014)* (see above, section 5.1) are fulfilled.

The parent/ patient's legally designated representative will be allowed as much time as wished to consider the information, and the opportunity to question the investigator or other independent parties to decide whether they will participate in the study; and must have the appropriate information presented in an understandable way and in a format suitable to their needs.

10.1.2 Study Discontinuation and Closure

The study may be temporarily discontinued or prematurely terminated if the sponsor judges it necessary for medical, safety, regulatory, or other reasons consistent with applicable laws, regulations, and good clinical practice.

Circumstances that may warrant termination or suspension include, but are not limited to:

- Determination of unexpected, significant, or unacceptable risk to participants
- Demonstration of efficacy that would warrant stopping
- Insufficient compliance to protocol requirements
- Data that are not sufficiently complete and/or evaluable
- Determination of futility

Study site participation may be discontinued if the investigator, or the ethical review board of the study site judges it necessary for any reason.

10.1.3 Confidentiality and Privacy

The study staff will ensure that the participant´s anonymity is maintained. The participants will be identified only by a participant code on the eCRF and any electronic database.

All documents will be stored securely and only accessible by study staff and authorized personnel.

Applicable regulations for storage, transmittal and disclosure of patient information will be always followed. The study will comply with the Data Protection Legislation.

Following formal admission to the study, patient data will be recorded in the hospital case record in the usual way including the circumstances of their entry to the study. Additionally, data will be held in case report forms (eCRF). These files will be identified by a study code, date of birth and participant code only.

Results of the study may be communicated at scientific meetings and will contribute to the scientific literature. At no time, will this be done in such a way that an individual patient may be identified.

10.1.4 Quality Assurance and Quality Control

The designee will implement and maintain quality control and quality assurance procedures with written standard operating procedures to ensure that the study is conducted and data are generated, documented, and reported in compliance with the protocol, ICH GCP, and applicable regulatory requirements.

QC procedures will be implemented beginning with the paper based primary study documentation that will be entered into the electronic data entry system by generating data QC checks on the database (edit checks).

Training for the study will be provided at the Study investigator's meeting (if applicable) and for site personnel prior to the initiation of study

The investigational site will provide direct access to all trial related sites, source data/documents, and reports.

Serious Breaches

A serious breach is defined as "A breach of GCP if applicable or the study protocol which is likely to effect to a significant degree –

(a) The safety or physical or mental integrity of the subjects of the study; or
(b) The scientific value of the study.

Any serious breaches will be notified to the competent Regulatory Authority according to applicable legislation.

Study file

The Investigator must maintain confidential all study documentation and take measures to prevent accidental or premature destruction of these documents. It is recommended that the Investigator retain the study documents at least twenty-five years after the completion or discontinuation of the Clinical Trial.

10.2. ABBREVIATIONS

AE	Adverse Event
AR	Adverse reaction
AUC	Area under the curve - or under the receiver operating characteristic curve (ROC)
BE	Base excess
BP	Blood Pressure
bpm	beats per minute
BW	Body Weight
CI	Confidence Interval
CMV	Continuous mandatory ventilation
CMP	Clinical Monitoring Plan
CPAP	Continuous positive airway pressure
CPC	Clinical Publication Committee
CRF	Case Report Form
CRP	C reactive protein
CRT	Capillary refill time
cUS	Cranial ultrasound
DSMB	Data Safety Monitoring Board
EDC	Electronic Data Capture

EC	Ethics Committee
Echo-D	Echocardiography-Doppler
eCRF	Electronic Case Report Form
EFCNI	European Foundation for the Care of Newborn Infants
EMA	European Medicines Agency
EudraCT	European Union Drug Regulating Authorities Clinical Trials
EoT	End of Treatment
GA	Gestational Age
GCP	Good Clinical Practice
Hb	Haemoglobin
HFNC	High flow nasal cannula
HFOV	High frequency oscillatory ventilation
HULP	La Paz University Hospital
ICD	Informed Consent Document(s)
ICH	International Conference on Harmonisation
IMP	Investigational Medicinal Product
ITT	Intention-To-Treat
IVH	Intraventricular haemorrhage
MABP	Mean Arterial Blood Pressure
MedDRA	Medical Dictionary for Regulatory Activities
NICU	Neonatal Intensive Care Unit
NIRS	Near-Infrared Spectroscopy
NS	Normal saline
OR	Odds Ratio
pCO2	Partial pressure of carbon dioxide
PD	Pharmacodynamic(s)
PDA	Patent Ductus Arteriosus
PI	Principal Investigator
PK	Pharmacokinetic(s)
PMA	postmenstrual age
RCT	Randomised controlled trial
ROC	Receiver operating characteristic
ROP	Retinopathy of prematurity
SAE	Serious adverse event
SAR	Serious adverse reaction
RVO	Right Ventricular Output
SaO2	Arterial oxygen saturation
SAE	Serious Adverse Event
SAR	Serious adverse drug reaction
SERMAS	Servicio Madrileño de Salud (Madrid, Spain)
SNAPPE-II	Score for Neonatal Acute Physiology Perinatal Extension
SoA	Schedule of Activities
SOP	Standard Operating Procedure
SpO2	Pulse oximetry
SUSAR	Suspected unexpected serious adverse reaction
SVC	Superior Vena Cava
TMF	Trial Master File
TSC	Trial Steering Committee

UCICEC	Central Research and Clinical Trials Unit
UCICEC-HULP	Central Research and Clinical Trials Unit at La Paz University Hospital

11. REFERENCES

1. Plomgaard AM, Hagmann C, Alderliesten T, Austin T, van Bel F, Claris O, et al. Brain injury in the international multicenter randomized SafeBoosC phase II feasibility trial: cranial ultrasound and magnetic resonance imaging assessments. Pediatr Res. 2016 Mar;79(3):466-72. PubMed PMID: 26571218. Pubmed Central PMCID: 4823642.

2. Brouwer MJ, van Kooij BJ, van Haastert IC, Koopman-Esseboom C, Groenendaal F, de Vries LS, et al. Sequential cranial ultrasound and cerebellar diffusion weighted imaging contribute to the early prognosis of neurodevelopmental outcome in preterm infants. PloS one. 2014;9(10):e109556. PubMed PMID: 25329772. Pubmed Central PMCID: 4203729.

3. Horsch S, Muentjes C, Franz A, Roll C. Ultrasound diagnosis of brain atrophy is related to neurodevelopmental outcome in preterm infants. Acta paediatrica. 2005 Dec;94(12):1815-21. PubMed PMID: 16421044.

4. Hyttel-Sorensen S, Pellicer A, Alderliesten T, Austin T, van Bel F, Benders M, et al. Cerebral near infrared spectroscopy oximetry in extremely preterm infants: phase II randomised clinical trial. BMJ. 2015 Jan 05;350:g7635. PubMed PMID: 25569128. Pubmed Central PMCID: 4283997.

5. Kluckow M, Evans N. Low superior vena cava flow and intraventricular haemorrhage in preterm infants. Arch Dis Child Fetal Neonatal Ed. 2000 May;82(3):F188-94. PubMed PMID: 10794784. Pubmed Central PMCID: 1721081. Epub 2000/05/05. eng.

6. Osborn DA, Evans N, Kluckow M. Hemodynamic and antecedent risk factors of early and late periventricular/intraventricular hemorrhage in premature infants. Pediatrics. 2003 Jul;112(1 Pt 1):33-9. PubMed PMID: 12837865. Epub 2003/07/03. eng.

7. Hunt RW, Evans N, Rieger I, Kluckow M. Low superior vena cava flow and neurodevelopment at 3 years in very preterm infants. Journal of Pediatrics. 2004 Nov;145(5):588-92. PubMed PMID: ISI:000225086800009. English.

8. Osborn DA, Paradisis M, Evans N. The effect of inotropes on morbidity and mortality in preterm infants with low systemic or organ blood flow. Cochrane Database Syst Rev. 2007 (1):CD005090. PubMed PMID: 17253539. Epub 2007/01/27. eng.

9. Pellicer A, Valverde E, Elorza MAD, Madero R, Gaya F, Quero J, et al. Cardiovascular support for low birth weight infants and cerebral hemodynamics: A randomized, blinded, clinical trial. Pediatrics. 2005 Jun;115(6):1501-12. PubMed PMID: ISI:000229504800007. English.

10. Pellicer A, Bravo MC, Madero R, Salas S, Quero J, Cabanas F. Early systemic hypotension and vasopressor support in low birth weight infants: impact on neurodevelopment. Pediatrics. 2009 May;123(5):1369-76. PubMed PMID: 19403504. Epub 2009/05/01. eng.

11. Bravo MC, Lopez-Ortego P, Sanchez L, Riera J, Madero R, Cabanas F, et al. Randomized, Placebo-Controlled Trial of Dobutamine for Low Superior Vena Cava Flow in Infants. J Pediatr. 2015 Sep;167(3):572-8 e1-2. PubMed PMID: 26116470.

12. Bravo MC, Lopez-Ortego P, Sanchez L, Madero R, Cabanas F, Koch A, et al. Validity of biomarkers of early circulatory impairment to predict outcome: An analysis in retrospect. Front Pediatr 2019.

13. Tuttle RR, Mills J. Dobutamine: development of a new catecholamine to selectively increase cardiac contractility. Circ Res. 1975 Jan;36(1):185-96. PubMed PMID: 234805.

14. Ruffolo RR, Jr., Spradlin TA, Pollock GD, Waddell JE, Murphy PJ. Alpha and beta adrenergic effects of the stereoisomers of dobutamine. J Pharmacol Exp Ther. 1981 Nov;219(2):447-52. PubMed PMID: 6270308.

15. Stopfkuchen H, Schranz D, Huth R, Jungst BK. Effects of Dobutamine on Left-Ventricular Performance in Newborns as Determined by Systolic-Time Intervals. European Journal of Pediatrics. 1987 Mar;146(2):135-9. PubMed PMID: ISI:A1987G301600006. English.

16. Stopfkuchen H, Queisser-Luft A, Vogel K. Cardiovascular responses to dobutamine determined by systolic time intervals in preterm infants. Crit Care Med. 1990 Jul;18(7):722-4. PubMed PMID: 2364711. Epub 1990/07/01. eng.

17. Devictor D, Verlhac S, Pariente D, Huault G. Hemodynamic effects of dobutamine in asphyxiated newborn infants. Arch Fr Pediatr. 1988 Aug-Sep;45(7):467-70. PubMed PMID: 3202670. Epub 1988/08/01. Effets hemodynamiques de la dobutamine chez le nouveau-ne asphyxie. fre.

18. Martinez AM, Padbury JF, Thio S. Dobutamine pharmacokinetics and cardiovascular responses in critically ill neonates. Pediatrics. 1992 Jan;89(1):47-51. PubMed PMID: 1728020.

19. Robel-Tillig E, Knupfer M, Pulzer F, Vogtmann C. Cardiovascular impact of dobutamine in neonates with myocardial dysfunction. Early Hum Dev. 2007 May;83(5):307-12. PubMed PMID: 16982162. Epub 2006/09/20. eng.

20. Mahoney L, Shah G, Crook D, Rojas-Anaya H, Rabe H. A Literature Review of the Pharmacokinetics and Pharmacodynamics of Dobutamine in Neonates. Pediatr Cardiol. 2016 Jan;37(1):14-23. PubMed PMID: 26346024.

21. Osborn DA, Evans N, Kluckow M. Left ventricular contractility in extremely premature infants in the first day and response to inotropes. Pediatr Res. 2007 Mar;61(3):335-40. PubMed PMID: 17314693. Epub 2007/02/23. eng.

22. Greenough A, Emery EF. Randomized trial comparing dopamine and dobutamine in preterm infants. Eur J Pediatr. 1993 Nov;152(11):925-7. PubMed PMID: 8276025. Epub 1993/11/01. eng.

23. Hentschel R, Hensel D, Brune T, Rabe H, Jorch G. Impact on blood pressure and intestinal perfusion of dobutamine or dopamine in hypotensive preterm infants. Biol Neonate. 1995;68(5):318-24. PubMed PMID: 8835086. Epub 1995/01/01. eng.

24. Klarr JM, Faix RG, Pryce CJ, Bhatt-Mehta V. Randomized, blind trial of dopamine versus dobutamine for treatment of hypotension in preterm infants with respiratory distress syndrome. J Pediatr. 1994 Jul;125(1):117-22. PubMed PMID: 8021760. Epub 1994/07/01. eng.

25. Roze JC, Tohier C, Maingueneau C, Lefevre M, Mouzard A. Response to Dobutamine and Dopamine in the Hypotensive Very Preterm Infant. Archives of Disease in Childhood. 1993 Jul;69(1):59-63. PubMed PMID: ISI:A1993LL65300015. English.

26. Ruelas-Orozco G, Vargas-Origel A. Assessment of therapy for arterial hypotension in critically ill preterm infants. Am J Perinatol. 2000;17(2):95-9. PubMed PMID: 11023168. Epub 2000/10/07. eng.

27. Filippi L, Pezzati M, Poggi C, Rossi S, Cecchi A, Santoro C. Dopamine versus dobutamine in very low birthweight infants: endocrine effects. Archives of Disease in Childhood-Fetal and Neonatal Edition. 2007 Sep;92(5):367-71. PubMed PMID: ISI:000249239500012. English.

28. Osborn D, Evans N, Kluckow M. Randomized trial of dobutamine versus dopamine in preterm infants with low systemic blood flow. J Pediatr. 2002 Feb;140(2):183-91. PubMed PMID: 11865269. Epub 2002/02/28. eng.

29. Kluckow M, Evans N. Superior vena cava flow in newborn infants: a novel marker of systemic blood flow. Arch Dis Child Fetal Neonatal Ed. 2000 May;82(3):F182-7. PubMed PMID: 10794783. Pubmed Central PMCID: 1721083. Epub 2000/05/05. eng.

30. Osborn DA, Evans N, Kluckow M, Bowen JR, Rieger I. Low superior vena cava flow and effect of inotropes on neurodevelopment to 3 years in preterm infants. Pediatrics. 2007 Aug;120(2):372-80. PubMed PMID: 17671064. Epub 2007/08/03. eng.

31. Habib DM, Padbury JF, Anas NG, Perkin RM, Minegar C. Dobutamine pharmacokinetics and pharmacodynamics in pediatric intensive care patients. Crit Care Med. 1992 May;20(5):601-8. PubMed PMID: 1572184.

32. Berg RA, Padbury JF, Donnerstein RL, Klewer SE, Hutter JJ. Dobutamine pharmacokinetics and pharmacodynamics in normal children and adolescents. Journal of Pharmacology and Experimental Therapeutics. 1993 June 1, 1993;265(3):1232-8.

33. Berg RA, Donnerstein RL, Padbury JF. Dobutamine infusions in stable, critically ill children: pharmacokinetics and hemodynamic actions. Crit Care Med. 1993 May;21(5):678-86. PubMed PMID: 8482088.

34. Schwartz PH, Eldadah MK, Newth CJ. The pharmacokinetics of dobutamine in pediatric intensive care unit patients. Drug Metab Dispos. 1991 May-Jun;19(3):614-9. PubMed PMID: 1680627. Epub 1991/05/01. eng.

35. Pellicer A, Fernández R, Jullien V, Gleeson C, Bravo MC, López Ortego P, et al. Open-label, uncontrolled, multinational pilot study (Phase I-II) to investigate the role and pharmacokinetics of a new neonatal formulation of dobutamine in the treatment of haemodynamic insufficiency in the immediate postnatal period. Pediatric Research (2021) 89:981 – 986.

36. Subhedar NV, Shaw NJ. Dopamine versus dobutamine for hypotensive preterm infants. Cochrane Database Syst Rev. 2003 (3):CD001242. PubMed PMID: 12917901. Epub 2003/08/15. eng.

37. Aslan Y, Koca L, Mutlu M, Tekelioglu Y, Erduran E. Apopitotic effects of dopamine and dobutamine on neutrophils of premature neonates. Journal of Maternal-Fetal & Neonatal Medicine. 2011 Sep;24(9):1155-8. PubMed PMID: ISI:000294742100016. English.

38. Kluckow M, Seri I. Clinical presentations of neonatal shock: the VLBW infant during the first postnatal day. Hemodynamics and cardiology Neonatal questions and controversies Philadelphia: Saunders Elsevier. 2008:147-77.

39. Bauer P, Kohne K. Evaluation of experiments with adaptive interim analyses. Biometrics. 1994 Dec;50(4):1029-41. PubMed PMID: 7786985.

40. Barrington KJ, Janaillac M. Treating hypotension in extremely preterm infants. The pressure is mounting. Arch Dis Child Fetal Neonatal Ed. 2016 May;101(3):F188-9. PubMed PMID: 26785856.

41. Mielgo VE, Valls ISA, Lopez-de-Heredia JM, Rabe H, Rey-Santano C. Hemodynamic and metabolic effects of a new pediatric dobutamine formulation in hypoxic newborn pigs. Pediatr Res. 2017 Mar;81(3):511-8. PubMed PMID: 27886191.

42. Richardson DK, Corcoran JD, Escobar GJ, Lee SK, for The Canadian NICU Network, The Kaiser Permanente Neonatal Minimum Data Set Wide Area Network, et al. SNAP-II and SNAPPE-II: Simplified newborn illness severity and mortality risk scores. The Journal of Pediatrics. 2001;138(1):92-100.

43. Evans N. Functional echocardiography in the neonatal intensive care unit. In: Kleinman C, Seri I, editors. Hemodynamics and Cardiology Neonatology Questions and Controversies Philadelphia, PA: Saunders; 2008. p. 83-109.

44. Miletin J, Dempsey EM. Low superior vena cava flow on day 1 and adverse outcome in the very low birthweight infant. Arch Dis Child Fetal Neonatal Ed. 2008 Sep;93(5):F368-71. doi: 10.1136/adc.2007.129304. Epub 2007 Dec 18. PMID: 18089627.

45. Evans N, Kluckow M, Simmons M, Osborn D. Which to measure, systemic or organ blood flow? Middle cerebral artery and superior vena cava flow in very preterm infants. Arch Dis Child Fetal Neonatal Ed. 2002 Nov;87(3):F181-4. doi: 10.1136/fn.87.3.f181. PMID: 12390987; PMCID: PMC1721487.

46. O´Quigley J, Pepe M, Fisher L (1990). Continual reassessment method: a practical design for phase I clinical trials in cancer, Biometrics; 46 (1): 33-48.

47. Lee and Cheung (2009). Model calibration in the continual reassessment method, Clinical Trials; 6 (3): 227-238.

48. Lee and Cheung (2011). Calibration of prior variance in the bayesian continual reassessment method, Statistics in Medicine; 30 (17): 2081-2089.

49. Agresti A, Coull BA (1998)., Approximate is Better than “Exact” for Interval Estimation of Binomial Proportions, American Statistician; 52 : 119-126.

50. La Rosée K, Huntgeburth M, Rosenkranz S, Böhm M, Schnabel P. The Arg389Gly beta1-adrenoceptor gene polymorphism determines contractile response to catecholamines. Pharmacogenetics. 2004 Nov;14(11):711-6. doi: 10.1097/00008571-200411000-00001. PMID: 15564877.

51. Yogev D, Basheer M, Blotnick S, Caraco Y, Muszkat M. Effects of sex and the common ADRB1 389 genetic polymorphism on the hemodynamic response to dobutamine. Pharmacogenet Genomics. 2015 Nov;25(11):555-63. doi: 10.1097/FPC.0000000000000174. PMID: 26313487.

52. Mao YM, Liu ZQ, Chen BL, Guo D, Han CT, Yang LJ, Wang SY, Fan L, Zhou HH. Effect of 393T>C polymorphism of GNAS1 gene on dobutamine response in Chinese healthy subjects. J Clin Pharmacol. 2009 Aug;49(8):929-36. doi: 10.1177/0091270009337945. Epub 2009 Jun 19. PMID: 19542315.
